# Large socio-economic, geographic and demographic disparities exist in exposure to school closures

**DOI:** 10.1038/s41562-021-01087-8

**Published:** 2021-03-18

**Authors:** Zachary Parolin, Emma K. Lee

**Affiliations:** 1 Bocconi University Department of Social and Political Sciences, Milan, Italy; 2 Columbia University Center on Poverty and Social Policy, New York, NY USA

**Keywords:** Education, Science, technology and society

## Abstract

The coronovirus disease 2019 (COVID-19) pandemic has prompted many school districts to turn to distance or at-home learning. Studies are emerging on the negative effects of distance learning on educational performance, but less is known about the socio-economic, geographic and demographic characteristics of students exposed to distance learning. We introduce a U.S. School Closure and Distance Learning Database that tracks in-person visits across more than 100,000 schools throughout 2020. The database, which we make publicly accessible and update monthly, describes year-over-year change in in-person visits to each school throughout 2020 to estimate whether the school is engaged in distance learning. Our findings reveal that school closures from September to December 2020 were more common in schools with lower third-grade math scores and higher shares of students from racial/ethnic minorities, who experience homelessness, have limited English proficiency and are eligible for free/reduced-price school lunches. The findings portend rising inequalities in learning outcomes.

## Abstract (web summary)

This study introduces a public dataset that finds that school closures in the United States have been more common in schools with lower math scores and higher rates of students from racial minorities, who experience homelessness, and who have lower incomes.

## Main

The COVID-19 pandemic has prompted many school districts to turn to distance or at-home learning. While school closures are deemed necessary to prevent the spread of the coronavirus, they carry important consequences for children’s educational development. Recent studies have demonstrated, for example, that students are learning far less through distance learning than they would in a traditional face-to-face setting^1–6^. Reductions in test scores appear to be particularly steep for students with less-educated parents^7^.

In the United States, 48 states and Washington, D.C. mandated or recommended the closure of schools in April 2020, a month in which COVID-19 rapidly spread across the country^8^. However, beginning in September 2020, the start of the new academic year for most schools, state and local governments adopted vastly different approaches to distance learning. As a result, school closures were distributed much more unevenly across the United States from September through December in comparison with the previous spring. To date, little is known on how exposure to school closure and distance learning varies across students of different socio-economic backgrounds, races/ethnicities and pre-COVID educational performance. Measuring these disparities in exposure to school closure and distance learning is critical for understanding the potential widening of learning disparities in the United States.

This study introduces and analyses a U.S. School Closure and Distance Learning Database that tracks in-person visits to the vast majority of K–12 public schools in the United States from January 2019 through December 2020. Specifically, we measure year-over-year change in visits to each school throughout 2020 to determine whether the school is engaged in distance learning after the onset of the pandemic. In-person attendance estimates are measured using aggregated, anonymized mobile phone data released each month through SafeGraph. Validation checks presented within this study suggest that our projections of schools engaged in distance learning in a given month are consistent with alternative data sources and with school-specific reports of distance learning. Our dataset, made public for all researchers to use, provides (1) the estimated share of schools with at least a 50% year-over-year decline in in-person visits in a month (our threshold for labelling a school as ‘mostly closed’ or engaged in distance learning) and (2) the mean year-over-year change in in-person visits for schools within each school district, census tract, county and state for each month in 2020. For each location and month, we provide these estimates for all schools, for elementary schools only and for middle and high schools (approximately grades six and above). The database covers 94% of school districts spanning 98% of counties in the United States.

To analyse the socio-economic, geographic and demographic distribution of students exposed to distance learning, we combine the SafeGraph data with a large set of school-level indicators to measure how exposure to distance learning varies by third-grade math performance and the share of students who experience homelessness, have limited English proficiency, are eligible for free/reduced-price school lunches and are from racial/ethnic minorities. First, these indicators inform us of pre-COVID disparities in educational performance; we know, for example, that students attending schools with higher levels of poverty and lower average test scores are less likely to graduate from high school^9,10^, and that academic achievement is increasingly stratified across income levels and race/ethnicity^11–13^. Second, the indicators inform us of the characteristics of students whose educational experience is most likely to be disrupted by the COVID-19 pandemic. If the schools and students with the greatest pre-COVID disadvantages are also those most exposed to school closures and distance learning, inequalities in learning outcomes may worsen.

Our findings reveal large disparities in exposure to distance learning that threaten to exacerbate regional, racial and class-based divides in educational performance in the United States. We find that exposure to distance learning from September through December 2020 was more common among schools with lower third-grade math scores, a higher share of students experiencing homelessness, more students eligible for free/reduced-price lunches and from racial/ethnic minorities. The race/ethnicity and math score gaps are particularly striking: in October, 35% of white students were exposed to distance learning, compared with 52% of Black students, 60% of Hispanic students and 65% of Asian students, though these gaps narrowed somewhat as school closures became more widespread in December. Moreover, schools recording the lowest third-grade math scores prior to the pandemic were, on average, around 15 percentage points more likely to be closed during September to December 2020 relative to schools with average test scores.

Given evidence that school closures are detrimental to educational performance, particularly for students from disadvantaged backgrounds^2,7,14–19^, the large socio-economic, geographic and demographic disparities in exposure to distance learning suggest that the COVID-19 pandemic is likely to exacerbate inequalities in learning outcomes across the United States.

## Results

**Fig. 1:**
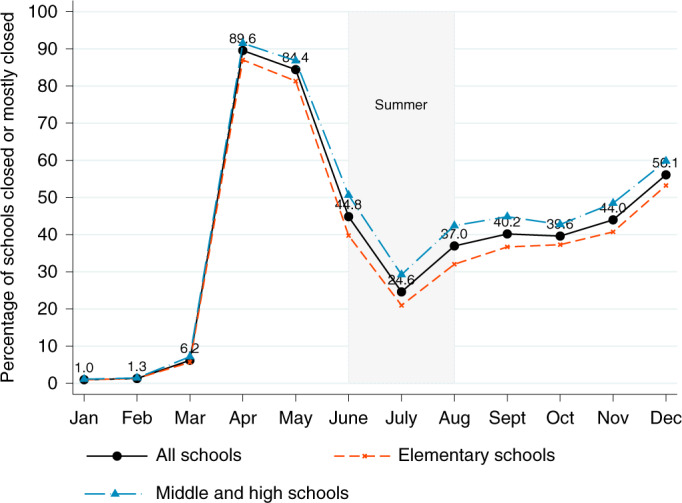
Share of schools closed or mostly closed. A school is ‘closed’ or ‘mostly closed’ if it experiences a year-over-year decline in in-person visits of at least 50% for a specific month. The sample includes 80,785 public schools per month in 2020.

To construct the U.S. School Closure and Distance Learning Database, we primarily use aggregated, anonymized mobile phone data from SafeGraph. We identify a school as ‘closed’ or ‘mostly closed’ if it experiences a 50% year-over-year decline in in-person visits during the given month. We discuss the dataset and validate its accuracy in the Methods section. Given that our data cover nearly all schools in the United States and are not randomly sampled from the full population of schools, we do not present confidence intervals for our estimates of school closures. Figure 1 presents the national trends in school closures from January through December 2020.

As expected, April 2020 features the peak of school closures. We estimate that 89.6% of all schools, including 92% of middle and high schools, turned to distance learning in April. This estimate corresponds closely with findings from the Census Household Pulse survey that 93% of families with children engaged in distance learning by summer^20^. In September, the start of the new academic year for many schools, an estimated 40.2% of all schools were closed. This subsequently climbed to 56.1% of schools in December 2020. Our findings suggest that middle and high schools were about 6.6 percentage points more likely than elementary schools to be engaged in distance learning in December. This is consistent with many schools’ desires to prioritize in-person learning for younger students.

**Fig. 2:**
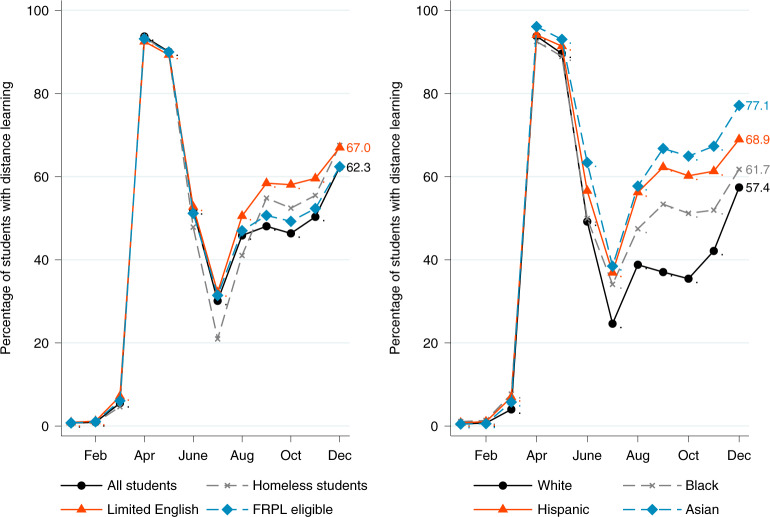
Share of students exposed to distance learning. A school is ‘closed’ or ‘mostly closed’ if it experiences a year-over-year decline in in-person visits of at least 50% for a specific month. The sample includes students attending a sample of 80,785 public schools in 2020. FRPL, free/reduced-price lunches.

Figure 2 switches focus from the characteristics of schools that are closed to the characteristics of students who are exposed to school closures. It also provides a look at socio-economic disparities in exposure to school closures. The left panel of Fig. 2 presents trends for all students, for students experiencing homelessness, for students with limited English proficiency and for students who are eligible for free/reduced-price lunches. While an estimated 56.1% of all schools were closed in December (from Fig. 1), Fig. 2 shows that 62.3% of all students were exposed to distance learning in December. This points to the fact that larger schools are more likely than smaller schools to have turned to distance learning, according to our database. In contrast, 67.5% of students experiencing homelessness and 67% of students with limited English proficiency were exposed to distance learning in December—both higher than the national average.

The right panel of Fig. 2 presents trends by race/ethnicity. Though students from each of the observed racial/ethnic categories faced similar rates of distance learning in April, the disparities widened throughout the autumn. In October, an estimated 35.4% of white students were exposed to distance learning, compared with 51.2% of Black students, 60.2% of Hispanic students, and 64.9% of Asian students. These disparities by race/ethnicity are comparable to estimates from Smith and Reeves^21^. By December, however, the share of all students exposed to distance learning increased, with white students seeing a particularly large increase to 57.4%. This rate, however, was still less than the rates of exposure for Black (61.7%), Hispanic (68.9%) and Asian (77.1%) students in December.

**Fig. 3:**
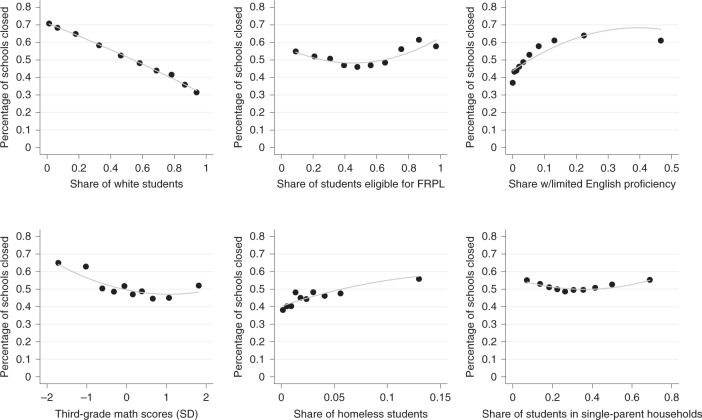
Share of schools closed or mostly closed by decile rank of given characteristic. Binned scatterplots where of the share of schools that are closed (50%+ year-over-year decline in in-person visits) among all schools in the specified decile. Average values from September to December 2020 for 80,785 public schools. FRPL, free/reduced-price lunches; SD, standard deviation.

Figure 3 narrows in on patterns of school closure across the distribution of each of our demographic and socio-economic characteristics. We average values over September through December 2020 to gain a more complete understanding of the disparities during the autumn term. Specifically, Fig. 3 bins schools according to their decile rank of the given characteristic on each *X* axis and the share of schools closed within that decile rank on the *Y* axis. Looking at the upper-left panel, for example, we see school closures across the distribution of the white share of students at a school. Among schools with the smallest share of white students (the first decile), around 70% of schools were closed, on average, during September through December. The subsequent deciles show a near-linear, inverse relationship between school closures and the share of white students at these schools. In schools with the highest share of white students (tenth decile), the rate of school closures is only 31%, less than half the rate of the bottom decile.

The upper-right panel shows a similar gradient with respect to the share of students with limited English proficiency. With the exception of the final decile, the share of school closures increases monotonically with the school’s share of non-native English speakers. The schools with the highest share of limited English proficiency were more than 20 percentage points more likely to be closed than schools with the lowest share of such students. Schools with the highest share of students eligible for free/reduced-price lunches were also more likely to be exposed to school closures from September through November.

Among the schools with the lowest math scores (bottom decile), an estimated 65% of schools were closed, on average, during September through December; among the second decile, around 64% of schools were closed. Both these rates are around 15 percentage points higher than schools with average levels of test scores. The bottom-middle panel presents school closures across the distribution of the share of K–12 students experiencing homelessness. Among the school districts with the lowest rates of student homelessness (bottom decile), just under 40% of schools were closed, on average. However, among schools with the highest rates of student homelessness, 57% were closed. Finally, the lower-right panel shows more muted variation across the distribution of the share of single parenthood across census tracts.

**Fig. 4:**
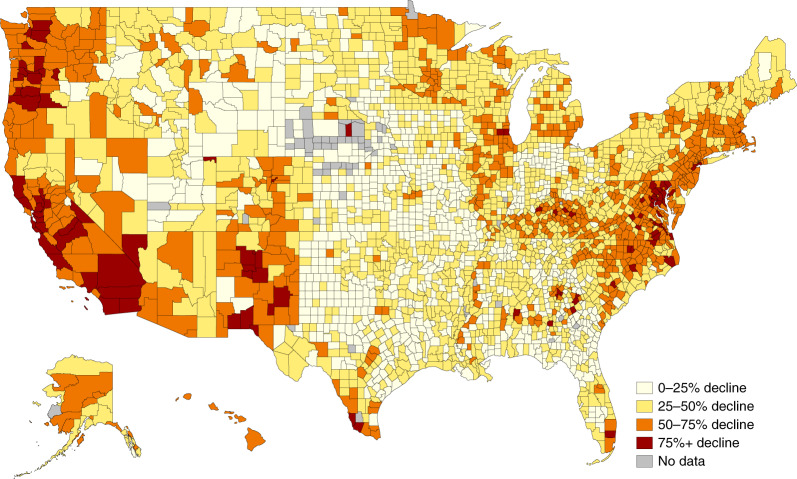
Mean year-over-year decline in in-person attendance among students per county. The sample includes 80,785 public schools with values averaged over September through December 2020.

Figure 4 visualizes the geographic disparities in school closures across the country. Specifically, it shows variation in the average year-over-year decline of in-person visits to schools in nearly every U.S. county. We again present averages from September through December 2020.

The darkest-shaded counties are those where schools, on average, saw declines of at least 75% in in-person visits from 2019 to 2020. These counties are concentrated on the West Coast in Washington, Oregon, California and Nevada, as well as the East Coast in Washington, D.C., Maryland, Massachusetts, New York and elsewhere. The counties with the smallest year-over-year declines in in-person visits tend to be concentrated in states across the Midwest and upper-Midwest, such as South Dakota, North Dakota, Wyoming, Montana, Iowa, Kansas and elsewhere. Data for all states, counties, census tracts and school districts are available in our public dataset.

## Discussion

After the onset of the pandemic, many schools turned to distance learning to prevent the spread of the virus. While deemed necessary for health and safety reasons, school closures likely carry costs related to learning outcomes. Recent studies have demonstrated that students exposed to distance learning have made ‘little or no progress while learning from home’ according to progressions in test scores, and that students from disadvantaged socio-economic backgrounds may face even steeper declines in learning outcomes^2,7,14^. Throughout the start of the 2020–2021 school year, not all students in the United States have been exposed to distance learning. This study provides descriptive evidence on the characteristics of students exposed to school closures during the COVID-19 pandemic.

Using anonymized mobile phone data to track year-over-year change in in-person visits to more than 100,000 schools throughout 2020, our findings reveal that closures throughout the autumn and winter of 2020 were more common in schools with lower third-grade math scores and higher shares of students who experience homelessness, have limited English proficiency, are eligible for free/reduced-price lunches and are from racial/ethnic minorities. In fact, only 35% of white students were exposed to distance learning in October 2020, compared with more than half of Black, Hispanic and Asian students. By December, rates of school closures spiked for all groups, though non-white students were still more likely to be learning remotely.

A number of factors likely explain these disparities. Most obviously, cities with larger, denser populations (which tend to have more racial/ethnic diversity) are perhaps at greater risk of transmitting COVID-19 than smaller, rural areas (which tend to be more white). Political differences in the likelihood that a state or local government orders schools to close may also factor into geographic variation in exposure to school closures. Regardless of cause, our findings show notable disparities in exposure to distance learning.

One should not infer from our findings that school closures, despite their estimated effects on educational performance, are unwarranted or that they necessarily do more harm than good. To the extent that schools are fertile grounds for the transmission of COVID-19, school closures may save lives, particularly in communities with more racial/ethnic minorities and/or lower incomes (the groups that this study finds are more likely to be exposed to school closures). The decision to turn to distance learning is undoubtedly difficult and is fraught with trade-offs; this study is not designed to address, and takes no position on, whether the costs of distance learning outweigh the benefits.

From the specific perspective of who is exposed to distance learning, however, our findings reveal clear disparities. Given that lower-income and/or non-white students already tend to fall behind academically, their greater exposure to school closures and distance learning may exacerbate socio-economic and racial/ethnic gaps in learning outcomes^12^. Moving forward, researchers can use our U.S. School Closure and Distance Learning Database to continue to investigate the consequences of school closures on education and socio-economic outcomes of relevance. The database is updated monthly and is freely accessible following the link provided in the Supplementary information section.

## Methods

### Data sources

To construct the U.S. School Closure and Distance Learning Database, we primarily use aggregated, anonymized mobile phone data from SafeGraph. SafeGraph uses Global Positioning System (GPS) data from around 10% of mobile devices (more than 40 million) in the United States to study mobility patterns and foot traffic to different businesses, schools and other public places. The SafeGraph sample of mobile devices closely corresponds to U.S. Census population counts by state (correlation of *r* = 0.977 between SafeGraph and Census counts across state) and county (*r* = 0.966). Similarly, strong correlations appear to exist between Census counts and the estimated racial/ethnic composition (*r* = 1.00), education group (*r* = 0.999) and 16-category household income bin (*r* = 0.997) of the SafeGraph sample^22^.

In the context of the COVID-19 pandemic, SafeGraph data have frequently been applied to measure the share of a community’s residents who appear to be social distancing or who appear to be engaged in full-time work^23–28^. For our purposes, we measure foot traffic to more than 100,000 schools across nearly every county in the United States to evaluate how the number of visits to each school in a given month in 2020 (say, December 2020) compares with the number of visits 12 months prior (December 2019). Data on the number of visits are released monthly and are available from January 2018 through December 2020 at the time of writing. Thus, for each month in 2020, we can track year-over-year change in the number of visits to each individual school in each month. Negligible year-over-year change in visit counts for a given school implies that the school is operating normally and is not engaged in large-scale distance learning; in contrast, a large year-over-year decrease in in-person visits implies that the school is engaged in distance learning.

In addition to measuring in-person visits to each school in each month, we identify the name of the school, its geographic location (including state, county, census tract and census block group) and the grade levels offered at the school. We incorporate a large selection of demographic and socio-economic covariates from alternative sources (discussed below) for each school to provide detailed data on the characteristics of students exposed to school closures and distance learning.

Our SafeGraph sample features data on 109,905 public and private schools in December 2020. Given that we have harmonized, comprehensive demographic data for all public schools, we exclude private schools and limit our analysis to the 80,785 public schools in the database. These schools span 12,727 school districts. The total number of public schools in the United States, according to the National Center for Education Statistics, is 98,469, and the total number of districts is around 13,584. That places our coverage rate of public schools at 82%, and coverage rate for districts at 94%. Compared with the schools in our SafeGraph data, the 18% of schools missing from our dataset have similar average enrolment sizes, shares of homeless students and eligibility for free/reduced-price lunches, but have students that are slightly more likely to be Black (average of 2.4 percentage points higher), Hispanic (0.4 percentage points) and Asian (0.7 percentage points). In a sensitivity check, we impute the missing in-person visit counts for schools not in our SafeGraph sample with the mean of other schools in the same district (based on the fact that schools within districts have heavily correlated changes in in-person visits). In doing so, we reach 93,314 schools for a coverage rate of 95%. The sensitivity check does not produce closure rates or socio-economic variance in closure rates that vary meaningfully from our primary analysis (in part due to the mean imputation). We thus only include the 80,785 schools for which we have observed data in our primary analyses.

### Key measures

Our primary indicator of interest is the year-over-year change in total visits to a school in a given month in 2020. For example, say that, in December 2019, there were 1,000 visits to a given elementary school, but in December 2020, that number fell to 200 visits. The year-over-year change for the given school is a decline of 80%. Using this indicator, we classify schools experiencing a year-over-year decline of at least 50% as being ‘closed’ or ‘mostly closed’. We use the word ‘closure’ in its de facto rather than de jure form: a school may not officially shut its doors or mandate distance learning, but if more than half the families appear to be engaged in distance learning, the school fits our definition of ‘school closure’ or large-scale distance learning that reduces in-person visits by at least 50% compared with 12 months prior. In the public database, we also provide estimates of the mean year-over-year decline in in-person visits and the share of schools with at least a 25% or 75% year-over-year decline in visits as alternative cutoff points.

### Demographic and socio-economic covariates

To provide detailed demographic and socio-economic information on students exposed to school closures and distance learning, we incorporate a large selection of school-level data on the characteristics of the students. Our primary data source is the Urban Institute’s Education Data Portal, which incorporates school-level information from the National Center for Education Statistics’ Common Core of Data (CCD), the Civil Rights Data Collection (CRDC) and the Integrated Public Use Microdata Series’ National Historical Geographic Information System (NHGIS)^29^. The CCD data provide information on whether the given school is an elementary, middle or high school (or unspecified), the number of students eligible for free/reduced-price school lunches and total student enrolment. The CRDC data provide breakdowns of enrolment by race and ethnicity, as well as the share of students with limited English proficiency. The NHGIS data provide the state, county, census tract and census block for each school (comparable to the geographic information we have for each school in our SafeGraph data). The data points are from the 2018–2019 school year. We merge these three external datasets into one, matching on each school’s unique identification number. We then merge this dataset with our SafeGraph data, matching on the state, county, census tract and census block of the school, as well as the name of the school (to account for the fact that some census blocks contain multiple schools).

Our second source of covariates is from the Opportunity Atlas (OA) dataset^30^, which includes the share of households with children headed by a single parent and the mean of third-grade math test scores in 2013 among schools in a given census tract. The math test scores available from the OA dataset are originally provided by the Stanford Education Data Archive. They represent the mean test scores of schools in the given census tract. In sensitivity checks, we also test results with more up-to-date (2017) estimates of math proficiency from the U.S. Department of Education’s EDFacts; the scores are strongly, positively correlated with their 2013 levels, but the EDFacts data feature more missing and censored values. We thus opt for the OA data in our primary analysis to maximize coverage.

Our third source is the National Center for Education Statistics^31^, which includes data on the share of students in a school district who experienced homelessness during the 2018–2019 school year. As detailed above, each of these indicators reflects pre-crisis disparities in economic and educational opportunities, but they also inform us of the characteristics of students whose educational experience is most likely to be disrupted by the COVID-19 pandemic. Students experiencing homelessness, for example, tend to struggle academically in non-pandemic times and are less likely to transition easily into home-based learning^32–34^. Similarly, students in high-poverty communities tend to have lower test scores and, further, may face greater resource challenges in preparing an adequate home-based learning environment when a school turns to distance learning.

### Validation checks

We present five validation checks to corroborate the accuracy of our school closure and distance learning estimates. First, the left panel of Supplementary Fig. 1 demonstrates that within-state changes in our estimates of school closures align closely with within-state changes in the share of families reporting distance learning in the Census Household Pulse Survey (CHPS) from April through November 2020 (*r* = 0.94).

Second, the right panel of Supplementary Fig. 1 also confirms that between-state means in our estimates of school closures from April through November align closely with state-level means from the CHPS (*r* = 0.75). Though the figure supports the consistency of the SafeGraph data with the CHPS data at the state level, we note that it is less informative of our dataset’s accuracy of school closures at the sub-state level. We thus add three validation checks for the school and school district estimates.

The third validation test is a manual cross-check of 102 schools in September (two from each state and Washington, D.C.) to compare our classification of whether the school is engaged in distance learning with online evidence from the school’s communication channels (primarily their websites). To select the schools to be cross-checked, we sorted schools by state and zip code, then selected the top two elementary schools listed for each state. For 91 of the 102 schools, we found supporting evidence on the school’s or school district’s website to confirm our classification of whether the school is mostly closed. For 10 of the 102 schools, we could not find evidence either way to support our classification of the school. For 1 of the 102 schools, we found evidence that contradicted our classification (the website of an elementary school in Aeia, Hawaii, suggests that it is operating in-person, while our data suggest that visits in September 2020 dropped by 70% from 12 months before).

Fourth, we also cross-check our results with Education Week’s (EW) manually coded school closure status of more than 907 school districts in September. Specifically, EW checked the websites of the 907 districts to assess whether the districts were reported to be learning ‘in-person’, under a ‘hybrid/partial’ scheme, or ‘fully remote’. Put differently, EW measures the district policies, whereas our data are meant to capture the actual change in average in-person visits to schools within a district (if some parents opt not to send their students to school despite the school’s being open for in-person learning, this would register in our data but not in the EW classification).

The districts that EW identifies as ‘fully remote’ overlap closely with our 50% decline in in-person visits benchmark. Specifically, Supplementary Fig. 2 shows the cumulative share of school districts in each of the EW categories (*Y* axis) across the distribution of decline in in-person visits (*X* axis). The results show that 80% of school districts with at least a 50% decline in in-person visits (according to our SafeGraph data) are labelled by EW as ‘fully remote’. The other 20% of districts meeting this benchmark are primarily in the ‘hybrid/partial’ group. Additionally, 73% of all districts that EW classifies as ‘fully remote’ meet the 50% cutoff according to our SafeGraph data. These results thus suggest that the 50% in-person visits cutoff effectively captures districts that have explicitly stated that they were fully remote; however, it does not capture all such districts (a minority of remote schools fall below the cutoff), nor does it exclusively capture such schools (some districts meeting this benchmark are classified as ‘hybrid/partial’). The evidence also suggests that a 25% year-over-year change benchmark captures the majority of districts in each of the three categories, whereas a 75% year-over-year change benchmark captures very few schools in general. Nonetheless, we include both benchmarks, as well as the mean year-over-year change in in-person visits, in our public dataset so that researchers have access to each of the benchmarks.

Fifth, we assess the intra-class correlation of the mean year-over-year change in in-person visits among schools within the same school district and grade level (elementary versus middle/high). Given that schools within the same district and grade level are generally subject to the same school closure policies, we expect to see a strong, positive correlation among the values for such schools. Indeed, the intra-class correlation is 0.73 in December 2020, suggesting that this is generally the case. Together, the five validation checks suggest that our estimates of school closures tend to be accurate, but do contain the possibility of measurement error.

### Limitations

As our data are measured from mobile phone usage, between-school comparisons could reflect differences in the likelihood that a parent, teacher or student arriving at a school uses a ‘smart phone’ (in addition to basic demographic factors, such as differences in population size). This explains why we do not measure differences between schools and instead evaluate year-over-year change for each individual school. It is possible that within-school changes in mobility behaviour over time affect our measures of change in in-person attendance. For example, if parents drove children to school in 2019 but began sending children to school by bus in 2020, the data may register a decrease in year-over-year visits (as parents are more likely to use mobile phones) and overstate the extent of distance learning. However, the opposite is more likely to be true (parents driving children in 2020 rather than sending by bus, where students may be more directly exposed to the virus), and if so, the consequence would be that the data do not identify the school as closed, which would be accurate given that the children are, indeed, attending school in this scenario. Alternatively, if the economic crisis forces families to adjust their cell phone plans, our data may overstate the extent of declines in in-person visits to schools.

In other scenarios, we may understate the extent of school closures if, as one example, students (and/or their parents) still travel to school every day to pick up school-provided meals, but then promptly return home. Such a scenario could introduce error in our estimates of in-person visits. Nonetheless, validation checks, as well as the descriptive evidence, provide confidence that the data tend to accurately represent the situation of the 100,000+ schools in the dataset.

### Analysis

Given that little is known to date regarding the socio-economic, geographic and demographic distribution of exposure to distance learning, this study primarily focuses on providing this descriptive evidence. Specifically, we show trends in school closures from January through December 2020 and provide a detailed breakdown of the types of students who are most likely to be learning from home. In future analyses, researchers can use our database to assess other types of disparity, such as access to stable internet connections, and to analyse the consequences of distance learning on employment, psychological wellbeing and other outcomes of interest.

### Reporting summary

Further information on research design is available in the Nature Research Reporting Summary linked to this article.

## Supplementary information

Supplementary information Supplementary Figs. 1 and 2.

Reporting summary

Peer review information

## Data Availability

We have made the U.S. School Closure and Distance Learning Database publicly available for researchers. It can be accessed at https://osf.io/tpwqf/. School-level data (beyond the level of school district) can be obtained by gaining approval to use SafeGraph’s data and then contacting the corresponding author.

## Code availability

Custom code that creates the descriptive statistics presented in this study can be accessed at https://osf.io/tpwqf/.
