# Supplementary material for: Large socio-economic, geographic and demographic disparities exist in exposure to school closures
Source: Nat Hum Behav. 2021 Mar 18;5(4):522–8. doi: 10.1038/s41562-021-01087-8 (PMC8060162; doi:10.1038/s41562-021-01087-8)

nature portfolio

https://doi.org/10.1038/s41562-021-01087-8

Supplementary information

Large socio-economic, geographic and demographic disparities exist in exposure to school closures

In the format provided by the authors and unedited

Supplementary Figures for:

Large Socio-Economic, Geographic, and Demographic Disparities Exist in Exposure to School Closures

Supplementary Figure 1: Within-state (left panel) and between-state (right panel) variation throughout 2020 in school closures (estimated from SafeGraph data) versus share of families reporting that their child is engaged in distance learning in Census Household Pulse Survey. r=0.94 and r=0.75, respectively. Share of families reporting children engaged in distance learning from Census Household Pulse Survey. Y-Axis represents share of schools closed in given state and month from SafeGraph estimates. Each point in left panel represents the value of the indicator for a given state-month minus the mean value of the indicator for the state across all months. Each point in right panel represents the mean value of the indicator for the state. December data for Census Household Pulse Survey not available at time of writing.

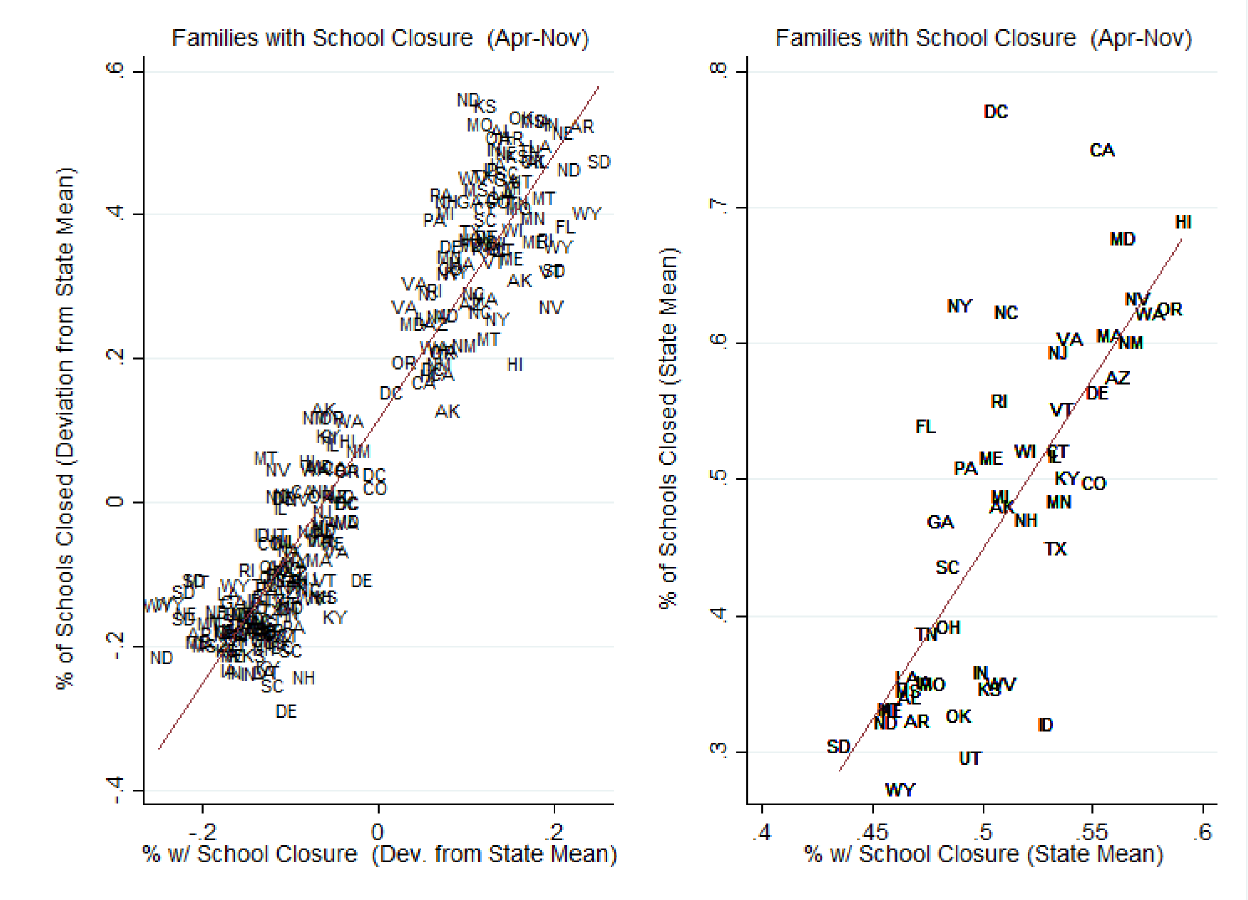

Supplementary Figure 2: Cumulative share of school districts across distribution of percent year-over-year decline in in-person visits, September 2020. EdWeek refers to Education Week analysis of 907 school districts in September 2020. EdWeek classified each of the 907 districts as beginning the school year with in-person learning, a hybrid/partial approach, or fully remote. Vertical lines represent potential cutoff points at 25% and 50% year-over-year declines. Y-axis represents the cumulative share of schools in the given EdWeek grouping that have a mean year-over-year change in in-person visits below the point on the X-axis.

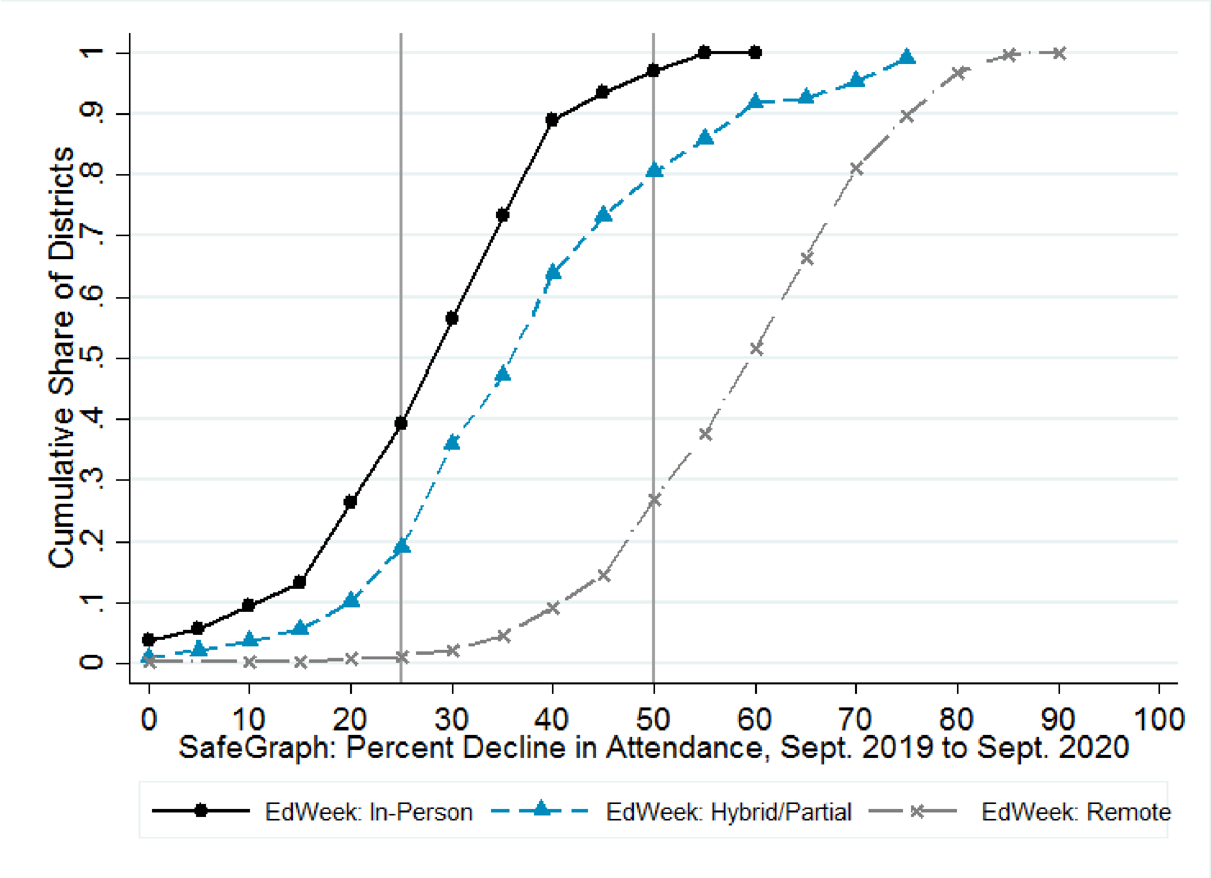

Supplement: Supplementary file 1 — Supplementary Figs. 1 and 2. [file 41562_2021_1087_MOESM1_ESM.pdf]
