# Supplementary material for: Large socio-economic, geographic and demographic disparities exist in exposure to school closures
Source: Nat Hum Behav. 2021 Mar 18;5(4):522–8. doi: 10.1038/s41562-021-01087-8 (PMC8060162; doi:10.1038/s41562-021-01087-8)
Supplement: Supplementary file 2 — Reporting summary [file 41562_2021_1087_MOESM2_ESM.pdf]

nature research

Corresponding author(s): Zachary Parolin
Last updated by author(s): 02/22/2021

# Reporting Summary

Nature Research wishes to improve the reproducibility of the work that we publish. This form provides structure for consistency and transparency in reporting. For further information on Nature Research policies, see our Editorial Policies and the Editorial Policy Checklist.

## Statistics

For all statistical analyses, confirm that the following items are present in the figure legend, table legend, main text, or Methods section.

| n/a | Confirmed | |
|---|---|---|
| ☐ | ☒ | The exact sample size (*n*) for each experimental group/condition, given as a discrete number and unit of measurement |
| ☐ | ☒ | A statement on whether measurements were taken from distinct samples or whether the same sample was measured repeatedly |
| ☒ | ☐ | The statistical test(s) used AND whether they are one- or two-sided<br>*Only common tests should be described solely by name; describe more complex techniques in the Methods section.* |
| ☒ | ☐ | A description of all covariates tested |
| ☒ | ☐ | A description of any assumptions or corrections, such as tests of normality and adjustment for multiple comparisons |
| ☒ | ☐ | A full description of the statistical parameters including central tendency (e.g. means) or other basic estimates (e.g. regression coefficient) AND variation (e.g. standard deviation) or associated estimates of uncertainty (e.g. confidence intervals) |
| ☒ | ☐ | For null hypothesis testing, the test statistic (e.g. *F*, *t*, *r*) with confidence intervals, effect sizes, degrees of freedom and *P* value noted<br>*Give P values as exact values whenever suitable.* |
| ☒ | ☐ | For Bayesian analysis, information on the choice of priors and Markov chain Monte Carlo settings |
| ☒ | ☐ | For hierarchical and complex designs, identification of the appropriate level for tests and full reporting of outcomes |
| ☒ | ☐ | Estimates of effect sizes (e.g. Cohen's *d*, Pearson's *r*), indicating how they were calculated |

*Our web collection on statistics for biologists contains articles on many of the points above.*

## Software and code

Policy information about availability of computer code

| | |
|---|---|
| Data collection | To construct the U.S. School Closure & Distance Learning Database, we primarily use aggregated, anonymized mobile phone data from SafeGraph. Access to SafeGraph data is free for researchers, but does require registration. Within the SafeGraph data, we identified schools using the specific NAICS code for K-12 schools. We then extracted SafeGraph Monthly Places Data for all K-12 schools for each month in 2019 through 2020. Please see the manuscript for further details on the SafeGraph data. For school-specific data, we merged in covariates from Urban Institute's Education Data Portal, which incorporates school-level information from the National Center for Education Statistics' Common Core of Data (CCD), the Civil Rights Data Collection (CRDC), and IPUMS' National Historical Geographic Information System (NHGIS). These data are free of charge and available at the Urban Institute's website. |
| Data analysis | We analyzed the data using Stata. After collecting the SafeGraph and school-specific data from Urban Institute, we exclusively rely on descriptive statistics in our analysis, as specified in full detail within the manuscript. |

For manuscripts utilizing custom algorithms or software that are central to the research but not yet described in published literature, software must be made available to editors and reviewers. We strongly encourage code deposition in a community repository (e.g. GitHub). See the Nature Research guidelines for submitting code & software for further information.

## Data

Policy information about availability of data

All manuscripts must include a data availability statement. This statement should provide the following information, where applicable:

- Accession codes, unique identifiers, or web links for publicly available datasets
- A list of figures that have associated raw data
- A description of any restrictions on data availability

We have made the U.S. School Closure & Distance Learning Database publicly available for researchers. It can be accessed at https://osf.io/tpwqf/. This data

aggregates school closure information to the county and state units of analysis for each month throughout 2020. We will update this database monthly. Within the manuscript, Figures 1-6 use the school closure data that we are making public.

# Field-specific reporting

Please select the one below that is the best fit for your research. If you are not sure, read the appropriate sections before making your selection.

☐ Life sciences ☒ Behavioural & social sciences ☐ Ecological, evolutionary & environmental sciences

For a reference copy of the document with all sections, see nature.com/documents/nr-reporting-summary-flat.pdf

# Behavioural & social sciences study design

All studies must disclose on these points even when the disclosure is negative.

| | |
|---|---|
| Study description | This descriptive study uses quantitative data from SafeGraph and Urban Institute 's Education Data Portal to estimate exposure to school closures and distance learning. |
| Research sample | The SafeGraph sample includes more than 40 million mobile device users across the United States. The SafeGraph data collected is anonymous at point of collection. As detailed in the manuscript, the mobile devices are dispersed randomly across the U.S. and align with Census population counts by county and state. We use the anonymized, aggregated data that SafeGraph provides free-of-charge to researchers. We then include all schools included in the SafeGraph sample, which is nearly all public schools in the U.S. |
| Sampling strategy | The sample includes mobile devices across the U.S. that agree to (or do not elect not to) share their anonymized location information with third-parties from whom SafeGraph collects data. The SafeGraph data collected is anonymous at point of collection. Data made available for researchers are anonymous and aggregated to the month and point-of-interest (schools in our case) levels. |
| Data collection | The data are from mobile devices across the U.S. that agree to (or do not elect not to) share their anonymized location information. The SafeGraph data collected is anonymous at point of collection. Data made available for researchers are anonymous and aggregated to the month and point-of-interest (schools in our case) levels. |
| Timing | Data collection is continuous among SafeGraph devices. The data made public are aggregates over the course of all days in a given month. |
| Data exclusions | Individuals without mobile devices or who elect not to share their data with third-party sources are excluded from the SafeGraph data universe. As discussed in the manuscript, non-participants do not appear to bias the data; SafeGraph mobile device counts are strongly and positively correlated with Census population counts across county and state. |
| Non-participation | Individuals without mobile devices or who elect not to share their data with third-party sources are excluded from the SafeGraph data universe. As discussed in the manuscript, non-participants do not appear to bias the data; SafeGraph mobile device counts are strongly and positively correlated with Census population counts across county and state. |
| Randomization | N/A |

# Reporting for specific materials, systems and methods

We require information from authors about some types of materials, experimental systems and methods used in many studies. Here, indicate whether each material, system or method listed is relevant to your study. If you are not sure if a list item applies to your research, read the appropriate section before selecting a response.

## Materials & experimental systems

| n/a | Involved in the study | |
|---|---|---|
| ☒ | ☐ | Antibodies |
| ☒ | ☐ | Eukaryotic cell lines |
| ☒ | ☐ | Palaeontology and archaeology |
| ☒ | ☐ | Animals and other organisms |
| ☒ | ☐ | Human research participants |
| ☒ | ☐ | Clinical data |
| ☒ | ☐ | Dual use research of concern |

## Methods

| n/a | Involved in the study | |
|---|---|---|
| ☒ | ☐ | ChIP-seq |
| ☒ | ☐ | Flow cytometry |
| ☒ | ☐ | MRI-based neuroimaging |
