# Supplementary material for: Large socio-economic, geographic and demographic disparities exist in exposure to school closures
Source: Nat Hum Behav. 2021 Mar 18;5(4):522–8. doi: 10.1038/s41562-021-01087-8 (PMC8060162; doi:10.1038/s41562-021-01087-8)
Supplement: Supplementary file 3 — Peer review information [file 41562_2021_1087_MOESM3_ESM.pdf]

natureresearch

Peer Review Information

Journal: Nature Human Behaviour
Manuscript Title: Large Socio-Economic, Geographic, and Demographic Disparities Exist in Exposure to School Closures
Corresponding author name(s): Zachary Parolin

Editorial Notes:

Reviewer Comments & Decisions:

Decision Letter, initial version:

7th January 2021

Dear Dr Parolin,

Thank you once again for your manuscript, entitled "National Database Reveals Large Socio-Economic, Geographic, and Demographic Disparities in Exposure to School Closures and Distance Learning in the US," and for your patience during the peer review process.

Your manuscript has now been evaluated by 5 reviewers, whose comments are included at the end of this letter. Although the reviewers find your work to be of interest, they also raise some important concerns. We are interested in the possibility of publishing your study in Nature Human Behaviour, but would like to consider your response to these concerns in the form of a revised manuscript before we make a decision on publication.

To guide the scope of the revisions, the editors discuss the referee reports in detail within the team, including with the chief editor, with a view to (1) identifying key priorities that should be addressed in revision and (2) overruling referee requests that are deemed beyond the scope of the current study. We hope that you will find the prioritised set of referee points to be useful when revising your study. Please do not hesitate to get in touch if you would like to discuss these issues further.

1) Please provide data at the school district and school level - in light of Reviewer 1's comments, it is critical for the use-value of your Resource to be able to provide access to data especially at the school district level (if not at the school level). Please address this concern - if there are legal and/or privacy constraints that prevent you from doing so, please detail these in your response to the reviewer's

comments and note them in your data availability statement as well. For any of the underlying data that cannot be publicly shared, please indicate in your data availability statement how interested researchers can obtain access (e.g., by contacting the corresponding author and with permission from SafeGraph).
2) Please clarify the composition of your school sample (in terms of public vs private) and address Reviewer 4's points regarding the composition of the 23% of schools that are not included in your database, as well as perform the sensitivity analysis recommended by the reviewer.
3) All additional validation and sensitivity analyses recommended by the reviewers should be performed, except in cases where they are unfeasible.
4) Please thoroughly address Reviewer 5's points 1 and 2 (point 1 also relates to Reviewer 1's request under point 1d).
5) Address all reviewer points regarding data presentation in the text, Figures and Tables.

Additionally, we have an editorial request for your revision, namely, that your analyses be updated to include data through to the end of December 2020 (or the latest available month in your database). This is important in order to ensure that, on eventual publication, your work presents an up-to-date (rather than outdated) picture of the state of school closures and distance learning in the country.

The reviewers also make several other suggestions that we feel are either best left for future research or are not advisable in light of journal policies:
* Reviewer 1's suggestion to include in your database (additional) private schools drawn from NCES.
* Reviewer 1's suggest to replace/supplement Opportunity Atlas achievement data with SEDA achievement data.
* Reviewer 2's request to shorten the results section by selecting the single best analysis to present.
* Reviewer 3's request to go beyond the current descriptive analyses.
* Reviewer 3's request you reorganise your manuscript. Our journal requirement is that the Methods follow the Results and Discussion and as such we ask that you retain this format. However, for readability please add a short summary of the data and methods at the beginning of your Results section.
* Reviewer 5's request that you examine internet access, given its importance for assessing inequality (this can be discussed as an aim for future research).

Finally, your revised manuscript must comply fully with our editorial policies and formatting requirements. Failure to do so will result in your manuscript being returned to you, which will delay its consideration. To assist you in this process, I have attached a checklist that lists all of our requirements. I have also attached a template manuscript file that exemplifies our policies and formatting requirements. If you have any questions about any of our policies or formatting, please don't hesitate to contact me.

In sum, we invite you to revise your manuscript taking into account all reviewer and editor comments. We are committed to providing a fair and constructive peer-review process. Do not hesitate to contact us if there are specific requests from the reviewers that you believe are technically impossible or unlikely to yield a meaningful outcome.

We hope to receive your revised manuscript within two weeks. We understand that the COVID-19 pandemic is causing significant disruption for many of our authors and reviewers. If you cannot send your revised manuscript within this time, please let us know - we will be happy to extend the

submission date to enable you to complete your work on the revision.

With your revision, please:

• Include a “Response to the editors and reviewers” document detailing, point-by-point, how you addressed each editor and referee comment. If no action was taken to address a point, you must provide a compelling argument. This response will be used by the editors to evaluate your revision and sent back to the reviewers along with the revised manuscript.

• Highlight all changes made to your manuscript or provide us with a version that tracks changes.

Please use the link below to submit your revised manuscript and related files:

[REDACTED]

Note: This URL links to your confidential home page and associated information about manuscripts you may have submitted, or that you are reviewing for us. If you wish to forward this email to co-authors, please delete the link to your homepage.

We look forward to seeing the revised manuscript and thank you for the opportunity to review your work. Please do not hesitate to contact me if you have any questions or would like to discuss these revisions further.

Sincerely,

Charlotte Payne
Editor
Nature Human Behaviour

Reviewer expertise:

Reviewer #1: Social and educational inequality

Reviewer #2: Economics of education

Reviewer #3: Economics of education

Reviewer #4: Spatial demography and inequality

Reviewer #5: Spatial demography and educational inequality

REVIEWER COMMENTS:

Reviewer #1:
Remarks to the Author:
The paper describes a novel data set that may be useful for understanding the patterns of school closure and their impacts on students and communities. Scholars of education, economics, and public health will find the data useful.

The data are derived from cell phone data showing the number of trips to each (public) school in the US, by month. The number of trips are compared to the number in the same school a year prior to determine where the number of trips has dropped substantially. This is a useful way of inferring school closures.

I think this is a valuable contribution. I have some suggestions to improve the data set and the paper. Some of these can be done quite quickly; others might wait for the next iteration of the data.

Data

1. Why not include private schools also? Private school data are available from the Private School Survey from NCES. I'm not sure if lat/long or address are available, but I think so. So it would seem easy to include private schools in the data set. Given anecdotal evidence and media reporting about lower school closure rates among private schools, it is likely that the patterns differ – this would be useful to know. And it would provide more complete population coverage within a county/state than public schools alone.

2. The data are made publicly available, which is great. But why report at the county level and not at the school and district level also? The paper implies that estimates are constructed for each school (change in cell traffic rate), but these school-level data are then aggregated to counties rather than being reported at the school level.

a. School districts are more relevant units than counties for much education research, and school closure policies are made, in many cases, at the district level. So it would be useful to provide district-level closure data (in addition to county and school).

b. When reporting data at the county or district or schools, consider including measures of the proportion of students (by demographic) in "closed" schools.

c. School-level data would allow researchers to aggregate in other ways and connect the closure data to other sources (ACS tract data, school data, etc).

d. Consider reporting estimated percentages reduction in cell traffic at the school (or district or county) level, rather than a binary indicator for whether the rate is above or below 50%; that would allow researchers to make different decisions about how to use the data.

e. Perhaps your agreement with SafeGraph and/or confidentiality concerns prevents some of the above, but to the extent that it does not, I would encourage reporting more detailed data and district-level data wherever possible.

3. Small note: I believe the SEDA data at https://edopportunity.org have more up to date and more detailed achievement data (by school, by grade, by year) than available at Opportunity Atlas. Data there are available by school, by district, and by county.

4. It might be useful to think about normalizing the cell count data by enrollment each year. If a school's enrollment declined by half from 2019-2020, this might register falsely as a closure. However, you may not have enrollment data from 2019 and 2020 to do this.

Validation:

The validation exercises in figure 6 are useful and compelling about the accuracy of the estimates at the state-month level. They are less informative about the validity of data at the county or school level.

Another validation is to see if closure patterns are consistent among schools of the same grade span within a school district – we might expect that all K-3 schools in a district would have the same pattern.

The exercise of picking 102 schools to manually check is useful, but the schools should have been picked at random within state. Picking 2 in the same zip code likely produces schools that are more similar to each other than would 2 schools picked at random, so there is not independent information in the 2 schools. Also, it would be good to show a correspondence table between the cell-data-inferred estimate and the manually-coded information, perhaps in a 2-x-2 table or using a ROC curve. If 95% of schools were closed the reported rate of correspondence is not as impressive as if 50% are closed.

Figures and Tables

Figure 1: maybe show share of students in distance learning on a second panel – to compare % of students to % of schools. I know this is in Figure 3, but might be useful to have the direct comparison in one figure.

Figure 2: are the state means enrollment-weighted? I think they should be. We want to know the proportion of students in closed schools, not the proportion of schools. Also, the figure would be more informative if it had more than three color values. And perhaps would be more useful based on district than county data.

Figure 3: When you show share of students, are you using 2019 enrollment and assuming it is unchanged in 2020? What are possible implications of this?

Figure 4: I don't find the figures classifying the x-variable by decile helpful. It abstracts from the actual proportions. It might be better to use the actual scale of the x-variable on the axis and use a binned scatterplot.

Table 1: Not very helpful – the same information is visible from the map; I would delete.

Reviewer #2:
Remarks to the Author:
The submitted paper is in general well-written and executed competently. It presents simple statistics from an exciting new database containing the monthly change in attendance rate (relative to the same month in 2019) for more than 100,000 primary and secondary schools in the US. Given that they also have socioeconomic and demographic schools' information, they can characterize the students and families more affected by the school closure. They show that the second semester's closure rate in 2020 is much higher for vulnerable students (measured in different ways).

I like the paper. It is simple, but also useful to better understand one relevant consequence of the covid. It is introducing a nice database that can be useful for future research.

1- Although the results are expected (the correlation between closure rate and vulnerability), it has a quite significant value to quantify this correlation. Moreover, it has a lot of value to build the database and make it public.

The results section can be shorter. You are showing the positive correlation between school closure and students' vulnerability in different ways. I suggest to pick the best way to show this correlation and dropping the other alternatives.

2- The analysis is correlational. Then it is not demanding in terms of statistical tools of sources of exogenous variation (to get causality). A concern that I have is the potential bias in the measure of school activity because this is built using mobile phone data. There are different reasons to be concerned. For example, it is reasonable to think that if parents lose their job because of covid they will not have the income to pay for their cellphone plan and the ones of their children.

In this regard, it is insufficient to test the accuracy of this empirical approach with 100 schools. This test should be done with at least 500/1000 schools randomly chosen (I do not see the difficulty in testing the accuracy of the approach for this number of schools).

3- In general, the use of statistics is adequate (it is a simple analysis, which is ok to me), but the analysis is weak where they try to distinguish between the inter-county and intra-county variation (page 11). One possibility is to use standard analysis of variance to calculate the between-group variation and the within-group variation.

4- I would present a histogram with the year-over-year change in total visits to a school for the full simple of schools and those labeled as mostly closed. This exercise can be useful to see how the analysis can be sensitive to the threshold's definition to be labeled as “mostly closed”. And it is also useful to show to the reader that there is nothing special about the chosen threshold.

5- For obvious reasons, there are not that many papers analyzing the closure of most of the schools in a country for an extended period. However, the paper can do a better job motivating these closures'

potential effect on future performance and students' lives. In particular, you may take a look at the papers that study the effect of school closure on dropouts and future grade retention, see for example (and the papers that they cited):

Hannum, Emily and Liu, Xiaoying and Wang, Fan, Estimating the Effects of Educational System Consolidation: The Case of China's Rural School Closure Initiative (February 16, 2018). Available at SSRN: https://ssrn.com/abstract=3140132

Grau, Nicolas, Daniel Hojman, and Alejandra Mizala. 2018. "School closure and educational attainment: Evidence from a market-based system." Economics of Education Review 65:1–17.

6- 100,000 schools sounds a big number, but how many schools are there is US? Is there any non-random selection of these schools?

Reviewer #3:
Remarks to the Author:
Thanks for giving me the chance to comment on the manuscript "National Database Reveals Large Socio-economic, Geographic, and Demographic Disparities in Exposure to School Closures and Distance Learning in the US".

The authors of this study build a school closure and distance learning database that tracks in-person attendance across more than 100,000 schools throughout 2020. This database allows the authors to estimate whether a school is engaged in distance learning based on year-over-year change in in-person attendance for each school. The authors take advantage of this database to describe the socio-economic, geographic, and demographic characteristics of schools and their students that are engaged in distance learning. They find that school closures in October 2020 (the start of the new academic year) are more common in schools with lower pre-COVID education results, measured by math scores in 3rd grade, with higher share of minority's students, with limited English proficiency, coming from families of lower socioeconomic status and living in single-parent families. In sum, the authors find that, as a result of the pandemic, inequalities in learning outcomes have increased, since studies have shown the negative effect of distance learning on educational performance vis a vis face-to-face learning.

Comment

The most important value added of the study is the construction of the database, which gathers very relevant information. The authors are careful to perform three validation checks to ensure that their estimates of schools engaged in distance learning are consistent with alternative data sources.
Also, the descriptive analysis gives relevant information to show how the effects of the COVID-19 pandemic are worsening the pre-COVID inequalities in US education. Indeed, family has always been a mediator between the school and the students, but never as decisive as today. Home is certainly not a school, but in distance learning it has been emulating it. To understand how critical this issue is, it is important to remember that to ensure children's equal rights, formal education has been organized as far as possible from the unequal student's family conditions, offering in exchange, a common experience in time, resources, curriculum, and space for sociability and study. Confining the school at

home significantly jeopardizes this elementary principle of equity. Therefore, in the case of the US, it is not only that children from low socioeconomic status families do not attend school, compared to children with more resources, but precisely those children for whom school is more relevant are not having that experience.

Besides putting together the database and provide a descriptive analysis I would like to see a greater use of the data to go beyond descriptive analysis and better understand the consequences of distance learning. It is necessary to provide empirical robust evidence to think about education policies aiming at closing these gaps.

I think it is better to organize the paper explaining first data sources and methods and then the results and their discussion

Reviewer #4:
Remarks to the Author:
The study "National Database Reveals Large Socio-Economic, Geographic, and Demographic Disparities in Exposure to School Closures and Distance Learning in the U.S." examines the association between school demographic and socioeconomic characteristics and distance learning based on school closure proxied by cell phone pings at school locations in over 100,000 schools in the United States. The authors found that school closures are associated with higher shares of racial/ethnic minority students, lower pre-COVID 19 test scores, and other measures of student socioeconomic and academic disadvantage. The study's use of cell phone data to answer an important question is quite novel. I offer several comments below.

Can the authors clarify whether they include private schools in their analysis. If they do include private schools, I suggest the authors testing the sensitivity of their results by focusing only on public schools. Some of the racial/ethnic and socioeconomic disparities may be driven by differences between the enrollment characteristics of public and private schools.

The authors state in a footnote on page 3 that their coverage rate is ``around 77 percent of all schools.' Can the authors describe or characterize the 23 percent of schools not included in the analysis?

The authors tested the sensitivity of their chosen closure cutoff by running the analyses using a 25 percent decline in attendance in addition to the 50 percent decline usedd in the main analysis. Why did the authors also not examine a higher cutoff, say, 75 percent?

Figure 3 examines student enrollment in closed schools as opposed to closed schools. I suggest also including enrollment in Table 2.

Figure 4 - top right graph - should be white students rather than white residents.

Many public school distance learning mandates or decisions are made at the school district level. Can the authors either include in their regression or justify why variation between district is not driving

results (in a similar way that states may be driving results in the example of Asian students on page 10).

References

Yang, T. C., Emily Choi, S. W., & Sun, F. (2020). COVID-19 cases in US counties: roles of racial/ethnic density and residential segregation. Ethnicity & health, 1-11.

Reviewer #5:
Remarks to the Author:
Review of NATHUMBEH-201213551

Thank you for the opportunity to review this novel and important paper. The database provided by the authors is valuable, as are the analyses. However, there are several concerns that need to be addressed before the paper is ready for publication.

1) The 50% threshold of year-over-year decline in in-person attendance to assign a school to distance learning seems arbitrary and potentially problematic. First, many schools and districts used a hybrid model with half of students attending on any given day to reduce density in schools. This scenario would reduce in-person attendance as measured by phone data by nearly half, but would not accurately indicate distance learning. Attending school in-person half-time allows teachers to come closer to the learning opportunities provided by full in-person attendance. Second, error in assignment based on the 50% threshold is likely correlated with the percent of students eligible for free lunch. Schools generally serve as the free lunch pick-up location for students attending school remotely. Schools with high poverty rates would be under-represented in those assigned to distance learning. The authors should take more advantage of the information provided in the continuous measure of percent decline in attendance compared to the previous year. For example, the authors could identify a representative group of schools that used a hybrid attendance model, learn what their year-over-year decline in attendance was, and create a range to indicate likely hybrid attendance. The authors could use a similar procedure to create a range for likely full distance learning schools. Alternatively, the authors could use the continuous measure of decline in attendance to quantify the relationship between attendance and their axes of inequality (e.g., race, ethnicity, free/reduced price lunch, ESL students).
2) The authors should address potential correlation between error in assignment to distance learning and the percent of students eligible for free lunch. It is difficult or misleading to compare their measures of inequality without addressing that bias.
3) Internet access is another important axis of inequality that would be valuable to examine in this paper.
4) The authors frame the study as measuring inequality in learning opportunities alone. However, school closure may be an important response to higher mortality in those areas. The authors could improve the paper by noting that the inequality in learning opportunities may represent a tradeoff with inequality in health/mortality risk.

Author Rebuttal to Initial comments

Response to the editors and reviewers:

Dear Editors and Reviewers: We greatly appreciate your comments and suggestions. We have addressed each below and we believe the manuscript now reads much better as a result. Given that this response is already lengthy, we will avoid a large summary of changes and proceed straight to our responses.

[Comments from the Editors:]

1) Please provide data at the school district and school level - in light of Reviewer 1's comments, it is critical for the use-value of your Resource to be able to provide access to data especially at the school district level (if not at the school level). Please address this concern - if there are legal and/or privacy constraints that prevent you from doing so, please detail these in your response to the reviewer's comments and note them in your data availability statement as well. For any of the underlying data that cannot be publicly shared, please indicate in your data availability statement how interested researchers can obtain access (e.g., by contacting the corresponding author and with permission from SafeGraph).

Response: We have clarified with SafeGraph that publicly providing district-level data for districts with at least two schools is possible without violating data sharing and privacy agreements. As such, we have now added the district-level estimates to our database (while still providing the Census tract, county, and state-level estimates for researchers who prefer those). Per the advice of reviewers, we now provide the average percent-change in in-person attendance for the district (or tract, county, state) in addition to the means for our binary 50% decline in attendance cutoff. This should provide researchers more flexibility when using the data and gets around concerns of selecting a semi-arbitrary cutoff (say, 25% or 50% decline in attendance) to identify school closures. We clarify these additions in the manuscript on pg. 4. We have also now detailed in the data availability statement that school-level data (beyond the level of school district) can be obtained by gaining approval to use SafeGraph’s data (free registration for researchers) and then contacting the corresponding author.

2) Please clarify the composition of your school sample (in terms of public vs private) and address Reviewer 4's points regarding the composition of the 23% of schools that are not included in your database, as well as perform the sensitivity analysis recommended by the reviewer.

Response: We have now clarified the composition of the schools in the SafeGraph sample. On pg. 11-12, we write that: “Our SafeGraph sample features data on 109,905 public and private schools in December 2020. Given that we have harmonized and comprehensive demographic data for all public schools, we exclude private schools and limit our analysis to the 80,785 public schools in the database. These schools span 12,727 school districts. The total number of public schools in the U.S., according to the National Center for Education Statistics, is 98,469, and the total number of districts is around 13,584. That places our coverage rate of public schools at 82 percent, and coverage rate for districts at 94 percent. Compared to the schools in our SafeGraph data, the 18 percent of schools missing from our dataset have similar average enrollment sizes, shares of homeless students, and free/reduced-price lunch eligibilities, but have students that are slightly more likely to be Black (average of 2.4 percentage points higher), Latino (0.4 percentage points), and Asian (0.7 percentage points).” We also discuss a sensitivity check in which we assess the effect of imputing missing in-person visits with the district-level mean of the schools with missing data. The results do not change, but to avoid adding potential measurement error, we exclude the imputation procedure from our primary analysis and only include the schools for which we have observed data.

3) All additional validation and sensitivity analyses recommended by the reviewers should be performed, except in cases where they are unfeasible.

Response: We have now added several sensitivity checks per requests of the reviewers. As discussed below in our responses to the reviewer suggestions, these include comparisons of our data to Education Week’s manually-collected school closure statuses for 907 districts in September (our estimates align closely with their classifications, as shown in Figure 6 and discussed below), assessments of intraclass correlations of the mean year-over-year change in attendance for schools within a given district (changes among schools within a district are correlated with an r=0.73), and the inclusion of different cutoff points and mean year-over-year changes in attendance within our public dataset. We discuss these in more detail below.

4) Please thoroughly address Reviewer 5's points 1 and 2 (point 1 also relates to Reviewer 1's request under point 1d).

Response: As discussed below in our response to Reviewer 5, we now include (1) the mean year-over-year change in in-person attendance for schools in our dataset to sidestep any concerns of an arbitrary cutoff point, (2) alternative cutoff points at 25% and 75% decline in attendance in our dataset, and (3) evidence in Figure 6 that the 50% cutoff that we prioritize effectively captures the vast majority (73%) of districts that Education Week identifies as “fully remote” while limiting overlap with the districts that Education Week identifies as “hybrid/partial”. With

respect to the reviewer’s concern of error related to free/reduced-price lunch (FRPL) status: we have investigated this by looking at whether year-over-year decline in the average “dwell time” (average time of which the mobile device is at the school) is higher for high-FRPL schools that are below our 50% in-person attendance cutoff. As discussed in our response below, however, we do not find sufficient evidence to support the reviewer’s concern. Nonetheless, we now acknowledge the concern in the manuscript on pg. 16 in our “Limitations” section. We discuss this in more detail below.

5) Address all reviewer points regarding data presentation in the text, Figures and Tables.

Response: We have now altered our tables, figures, and data presentation in line with the reviewers’ suggestions. The only exception is the reviewer who suggested we condense all figures/tables into one (though we did delete two extraneous tables and one figure to condense the findings section).

6) Additionally, we have an editorial request for your revision, namely, that your analyses be updated to include data through to the end of December 2020 (or the latest available month in your database). This is important in order to ensure that, on eventual publication, your work presents an up-to-date (rather than outdated) picture of the state of school closures and distance learning in the country.

Response: We have updated the manuscript to include data through the end of December 2020. Of course, we will also continue to update the public dataset each month to add in subsequent months’ estimates.

[The reviewers also make several other suggestions that we feel are either best left for future research or are not advisable in light of journal policies:]

* Reviewer 1's suggestion to include in your database (additional) private schools drawn from NCES.

Response: We agree with the editors’ judgment not to add additional private schools; data on such schools unfortunately not available in the Urban Institute’s Education Data Portal and its underlying datasets.

* Reviewer 1's suggest to replace/supplement Opportunity Atlas achievement data with SEDA achievement data.

Response: We agree with the editors' judgment not to address this request. The Opportunity Atlas data itself is based on the SEDA data and includes fewer missing/ranged values.

* Reviewer 2's request to shorten the results section by selecting the single best analysis to present.

Response: We have condensed the results section to streamline the analysis, but we agree with the editors' judgment of not cramming all findings into a single figure.

* Reviewer 3's request to go beyond the current descriptive analyses.

Response: We agree with the editors' judgment not to add in new analyses. Our hope is that our database prompts many new studies on the effects of school closures, but our goal here is to introduce the dataset and provide a rich initial description of who is exposed to school closures.

* Reviewer 3's request you reorganise your manuscript. Our journal requirement is that the Methods follow the Results and Discussion and as such we ask that you retain this format. However, for readability please add a short summary of the data and methods at the beginning of your Results section.

Response: We have added a short summary of data/methods at the beginning of the results section, but have kept the current format per the editorial guidelines.

* Reviewer 5's request that you examine internet access, given its importance for assessing inequality (this can be discussed as an aim for future research).

Response: We lack high-quality data on internet access at the local level. We agree that this is an opportunity for future research and have noted this now in our conclusion (p. 17).

Finally, your revised manuscript must comply fully with our editorial policies and formatting requirements. Failure to do so will result in your manuscript being returned to you, which will delay its consideration. To assist you in this process, I have attached a checklist that lists all of our requirements. I have also attached a template manuscript file that exemplifies our policies and formatting requirements. If you have any questions about any of our policies or formatting, please don't hesitate to contact me.

Response: We have reviewed the checklist and ensured that our manuscript meets the guidelines.

[Reviewer #1:]

The paper describes a novel data set that may be useful for understanding the patterns of school closure and their impacts on students and communities. Scholars of education, economics, and public health will find the data useful. The data are derived from cell phone data showing the number of trips to each (public) school in the US, by month. The number of trips are compared to the number in the same school a year prior to determine where the number of trips has dropped substantially. This is a useful way of inferring school closures.

I think this is a valuable contribution. I have some suggestions to improve the data set and the paper. Some of these can be done quite quickly; others might wait for the next iteration of the data.

1. Why not include private schools also? Private school data are available from the Private School Survey from NCES. I'm not sure if lat/long or address are available, but I think so. So it would seem easy to include private schools in the data set. Given anecdotal evidence and media reporting about lower school closure rates among private schools, it is likely that the patterns differ – this would be useful to know. And it would provide more complete population coverage within a county/state than public schools alone.

Response: Unfortunately, the detailed data for public schools that we have from the Urban Institute's Education Data Portal and its underlying datasets is not the same level detail as the data that we have for the private schools. The Urban data itself does not include private schools, and the NCES Private School Survey does not include much of the same level of detail. As such, we agree with the editors' judgment not to address this concern, as much as we'd like to be able to. We now clarify this on pg. 11-12.

2. The data are made publicly available, which is great. But why report at the county level and not at the school and district level also? The paper implies that estimates are constructed for each school (change in cell traffic rate), but these school-level data are then aggregated to counties rather than being reported at the school level. School districts are more relevant units than counties for much education research, and school closure policies are made, in many cases, at the district level. So it would be useful to provide district-level closure data (in addition to county and school).

Response: We agree with the reviewer that providing district-level data would be an important addition. We have confirmed with SafeGraph that we can provide such data for districts with at

least two schools. We have added the district-level data into our public datasets, and we now mention this in the manuscript in several places, the first of which is on pg. 3-4.

B / C. When reporting data at the county or district or schools, consider including measures of the proportion of students (by demographic) in “closed” schools. School-level data would allow researchers to aggregate in other ways and connect the closure data to other sources (ACS tract data, school data, etc).

Response: We agree with this request and have now added in data on the share of students (rather than share of schools) who are exposed to distance learning. Figures 2, 3, and 4 all present results displaying the share of students, rather than schools, who are exposed to distance learning. We still present the school-specific data in Figure 1 to provide readers with both sets of information.

D / E. Consider reporting estimated percentages reduction in cell traffic at the school (or district or county) level, rather than a binary indicator for whether the rate is above or below 50%; that would allow researchers to make different decisions about how to use the data. Perhaps your agreement with SafeGraph and/or confidentiality concerns prevents some of the above, but to the extent that it does not, I would encourage reporting more detailed data and district-level data wherever possible.

Response: We agree with this request and now include in our public dataset the mean year-over-year decline in in-person attendance among schools in a given district, tract, county, or state. This is in addition to the binary indicators of whether the rate is above/below 50%, which we still leave in the dataset for researchers who want to use that specific estimate.

3. Small note: I believe the SEDA data at https://edopportunity.org have more up to date and more detailed achievement data (by school, by grade, by year) than available at Opportunity Atlas. Data there are available by school, by district, and by county.

Response: We agree with the editors’ judgment not to address this request. The Opportunity Atlas data itself is based on the SEDA data and includes fewer missing/ranged values than the actual SEDA data.

4. It might be useful to think about normalizing the cell count data by enrollment each year. If a school’s enrollment declined by half from 2019-2020, this might register falsely as a closure. However, you may not have enrollment data from 2019 and 2020 to do this.

Response: The reviewer is correct that we do not have enrollment data from 2019-2020 to fulfill this request. The enrollment data are from 2018-2019. That said, we do not have sufficient reason to believe that a meaningful share of schools will see a 50% reduction in school enrollment over a single year. If this occurs, we expect it to be rare and not to meaningfully alter our overall findings.

* The validation exercises in figure 6 are useful and compelling about the accuracy of the estimates at the state-month level. They are less informative about the validity of data at the county or school level.

Response: We agree with the reviewer. The Pulse data do not have county or school-level data, so we cannot test the same exercise at lower levels than that of the state. Please note, though, that we have added in an additional sensitivity check comparing our estimates of school closures to Education Week's manual coding of 907 districts from September. These findings are discussed on pg. 14 and elsewhere in this response letter.

* Another validation is to see if closure patterns are consistent among schools of the same grade span within a school district – we might expect that all K-3 schools in a district would have the same pattern.

Response: We now add a measure the intraclass correlation of year-over-year declines in attendance among schools of a given grade type (elementary or middle/high school) within a school district. As detailed on pg. 16, the intraclass correlation is 0.73 -- adding further evidence that the data tend to be consistent with expectations (more specifically, that schools of a given grade within a given district tend to have similar year-over-year changes in in-person attendance). That the correlation is not stronger could reflect a combination of (1) some districts allowing school-level variation in closure or hybrid learning decisions, (2) within-district variation in extent to which parents decide to sent their children to school, even if open, or (3) measurement error among schools within a district.

* The exercise of picking 102 schools to manually check is useful, but the schools should have been picked at random within state. Picking 2 in the same zip code likely produces schools that are more similar to each other than would 2 schools picked at random, so there is not independent information in the 2 schools. Also, it would be good to show a correspondence table between the cell-data-inferred estimate and the manually-coded information, perhaps in a 2-x-2 table or using a ROC curve. If 95% of schools were closed the reported rate of correspondence is not as impressive as if 50% are closed.

Response: We understand the reviewer's concern and have added new data to our validation checks. Specifically, we now use an Education Week dataset of manually-checked closure status of 907 school districts across the U.S. in September. The EdWeek data coded schools as in-person, mixed/hybrid, or fully remote. We explain the results on pg. 15; as noted there, our results are consistent with EdWeek's classifications. Added to the 100 schools we manually cross-checked on our own, we now have more than 1,000 manual cross-checks as part of our validation analysis. This is in addition to the strong, positive correlations between our state/month-level estimates and those of the Census Pulse Household Survey (Figure 5).

* Figure 1: maybe show share of students in distance learning on a second panel – to compare % of students to % of schools. I know this is in Figure 3, but might be useful to have the direct comparison in one figure.

Response: Given that the data are already presented in what was Figure 3 (now Figure 2), we prefer not to add an extra figure (or panel) to show the same findings. This is especially true in light of other reviewer comments to condense the Findings section. To respect the spirit of the reviewer's request, however, we have moved the former Figure 3 (now Figure 2) to immediately follow Figure 1. This allows the reader to see the student-weighted estimates immediately after seeing the school estimates.

* Figure 2: are the state means enrollment-weighted? I think they should be. We want to know the proportion of students in closed schools, not the proportion of schools. Also, the figure would be more informative if it had more than three color values. And perhaps would be more useful based on district than county data.

Response: We have now updated the former Figure 2 (now Figure 3) per the reviewer's recommendations (and to reflect December 2020 data). We now weight the means by student enrollment (to reflect share of students rather than share of schools), and we have added a third category/color to capture counties with 75%+ decline in average in-person attendance. Unfortunately, we cannot map based on school district in this Stata mapping feature (or in comparable commands that we searched); we attempted to map by Census tract, but the outlines of tracts are so numerous that they dominate the figure. We instead stick to counties within this figure, as they provide a legible and clearly-delineated unit of analysis. Keep in mind, though, that researchers can still access more local data in our public-access datasets.

* Figure 3: When you show share of students, are you using 2019 enrollment and assuming it is unchanged in 2020? What are possible implications of this?

Response: Yes, we use the 2018-2019 enrollment data for our student-specific estimates. We do not have a compelling reason to believe that large between-school shifts in enrollment would have occurred between 2018-2019 and subsequent years in a way that will meaningfully affect our findings. To ensure greater clarity of our findings, however, we now note that we use the 2018-2019 data on pg. 13.

Figure 4: I don't find the figures classifying the x-variable by decile helpful. It abstracts from the actual proportions. It might be better to use the actual scale of the x-variable on the axis and use a binned scatterplot.

Response: We agree with the reviewer and have now replaced the former Figure 4 (now Figure 3) with a version that uses binned scatterplots for each of our variables of interest. This now provides the scale of the X-axis variable (ex: share of school that is white) across the X-axis, rather than simply the decile number.

* Table 1: Not very helpful – the same information is visible from the map; I would delete.

Response: We have deleted this table to streamline the analysis and avoid presenting overlapping information in the figures/tables.

[Reviewer #2:]

The submitted paper is in general well-written and executed competently. It presents simple statistics from an exciting new database containing the monthly change in attendance rate (relative to the same month in 2019) for more than 100,000 primary and secondary schools in the US. Given that they also have socioeconomic and demographic schools' information, they can characterize the students and families more affected by the school closure. They show that the second semester's closure rate in 2020 is much higher for vulnerable students (measured in different ways). I like the paper. It is simple, but also useful to better understand one relevant consequence of the covid. It is introducing a nice database that can be useful for future research.

1- Although the results are expected (the correlation between closure rate and vulnerability), it has a quite significant value to quantify this correlation. Moreover, it has a lot of value to build the database and make it public. The results section can be shorter. You are showing the positive correlation between school closure and students' vulnerability in different ways. I suggest to pick the best way to show this correlation and dropping the other alternatives.

Response: We have shortened the results section by, for example, deleting Tables 1 and 2 (which overlapped with information presented in the figures), the former Figure 4, and we

condensed some of the text. We have not condensed all findings to a single figure, but we hope the readers will now be able to navigate our results in a more efficient manner.

2- The analysis is correlational. Then it is not demanding in terms of statistical tools of sources of exogenous variation (to get causality). A concern that I have is the potential bias in the measure of school activity because this is built using mobile phone data. There are different reasons to be concerned. For example, it is reasonable to think that if parents lose their job because of covid they will not have the income to pay for their cellphone plan and the ones of their children.

Response: Indeed, such a scenario is possible, but we do not have evidence to suggest that families are closing their cellphone plans at such a rate to bias our results. Our validation checks suggest that our results operate as expected given the external data on school closures that is available. To accommodate the reviewer's concern, however, we now note this possibility on pg. 16-17.

In this regard, it is insufficient to test the accuracy of this empirical approach with 100 schools. This test should be done with at least 500/1000 schools randomly chosen (I do not see the difficulty in testing the accuracy of the approach for this number of schools).

Response: We have now added an additional validation check using an Education Week dataset of manually-checked closure status of 907 school districts across the U.S. in September.We explain the results on pg. 15; as noted there, our results are consistent with EdWeek's classifications. Added to the 100 schools we manually cross-checked on our own, we now have more than 1,000 manual cross-checks as part of our validation analysis. This is in addition to the strong, positive correlations between our state/month-level estimates and those of the Census Pulse Household Survey (Figure 5).

3- In general, the use of statistics is adequate (it is a simple analysis, which is ok to me), but the analysis is weak where they try to distinguish between the inter-county and intra-county variation (page 11). One possibility is to use standard analysis of variance to calculate the between-group variation and the within-group variation.

Response: We have decided to exclude this analysis from the revised version of our manuscript. In addition to this reviewer's concern, another reviewer had interpreted the analysis and figure as if school districts (compared to counties or states) were unimportant in shaping geographic variation in school closures. Instead, we simply meant to assess whether disparities exist within states and counties, in addition to across states and counties, but such an analysis is not necessary

for this paper. An added challenge is that these results vary by month (ex: September 2020 estimates look different than December 2020 estimates, which may look different than estimates in 2021 given ongoing changes in rates of school closures). Taking all of this into account, we decided to delete the figure and analysis and instead focus simply on providing the rich set of descriptives that are present in our other figures. To properly account for within- versus between-jurisdiction variation in closures requires a more detailed analysis and space to assess changes in these patterns by month. The decision to remove this analysis also meets the suggestion of other reviewers to condense our Findings section.

4- I would present a histogram with the year-over-year change in total visits to a school for the full simple of schools and those labeled as mostly closed. This exercise can be useful to see how the analysis can be sensitive to the threshold's definition to be labeled as “mostly closed”. And it is also useful to show to the reader that there is nothing special about the chosen threshold.

Response: A version of the author’s request is displayed in the new Figure 6. Specifically, it shows the share of school districts across the distribution of year-over-year changes in in-person attendance (divided into three categories within the EdWeek data from September, as this figure also serves as our cross-check with EdWeek data, as noted elsewhere in this review). This figure indeed shows that there is some (albeit relatively minor) overlap between districts that we identify as “closed” and districts that EdWeek identifies as “hybrid/remote” and “fully remote.” As we write on pg. 15: “The results show that 80 percent of school districts with at least a 50 percent decline in in-person attendance (according to our SafeGraph data) are labeled by EW as “fully remote”. The other 20 percent of districts meeting this benchmark are primarily the “hybrid/partial” group. Additionally, 73 percent of all districts that EW classifies as “fully remote” meet the 50 percent cutoff according to our SafeGraph data. The results thus suggest that the 50 percent in-person attendance cutoff effectively captures districts that have explicitly stated that they were fully remote; however, it does not capture all such districts (a minority of remote schools fall below the cutoff), nor does it exclusively capture such schools (some districts meeting this benchmark are classified as “hybrid/partial”)”. Note, as well, that we now include the mean year-over-year change in in-person visits into our public datasets, so researchers interested in the continuous variable can use it in their work (in addition to or as opposed to the binary indicators). If the editors believe an extra figure with a histogram should be added on top of what is already displayed, we are happy to include the figure below into the manuscript.

Figure: Histogram of year-over-year change in visits by school

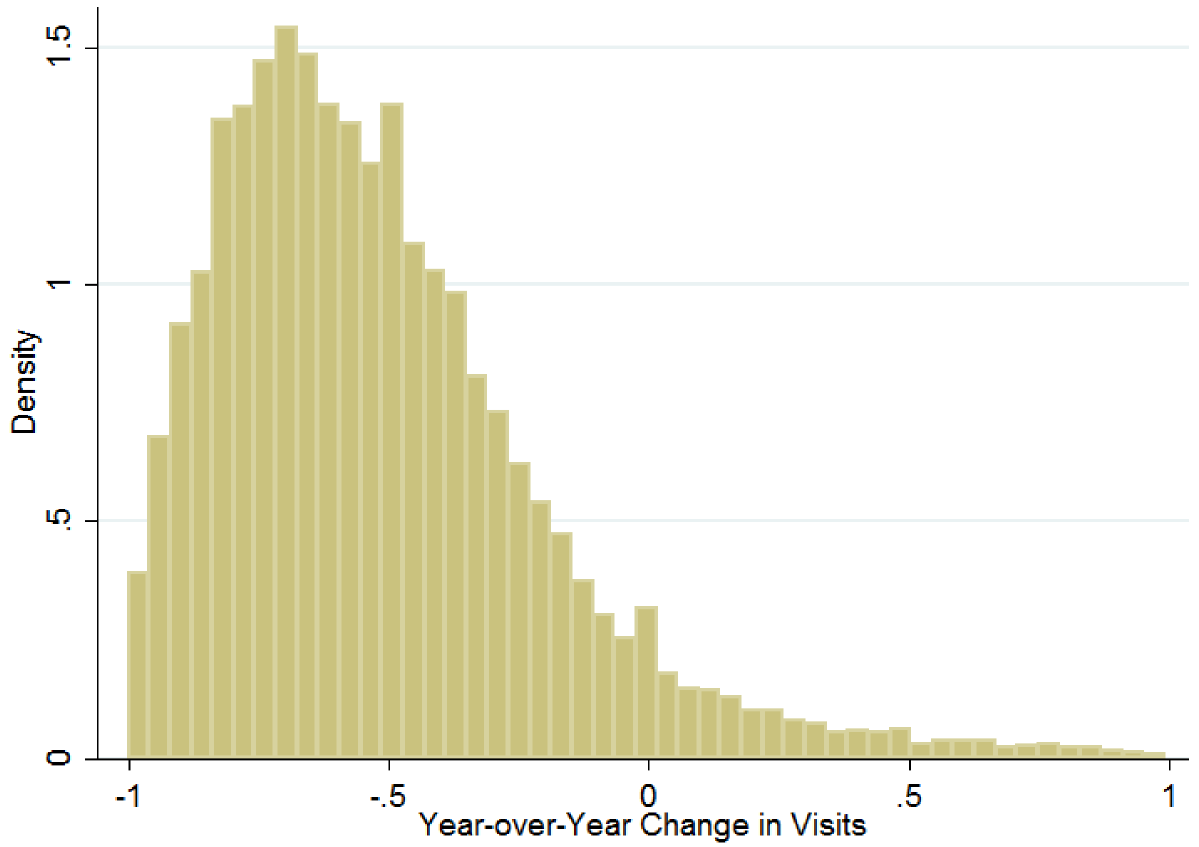

5- For obvious reasons, there are not that many papers analyzing the closure of most of the schools in a country for an extended period. However, the paper can do a better job motivating these closures' potential effect on future performance and students' lives. In particular, you may take a look at the papers that study the effect of school closure on dropouts and future grade retention, see for example (and the papers that they cited):

Hannum, Emily and Liu, Xiaoying and Wang, Fan, Estimating the Effects of Educational System Consolidation: The Case of China's Rural School Closure Initiative (February 16, 2018). Available at SSRN: https://ssrn.com/abstract=3140132

Grau, Nicolas, Daniel Hojman, and Alejandra Mizala. 2018. “School closure and educational attainment: Evidence from a market-based system.” Economics of Education Review 65:1–17.

Response: We appreciate the reviewer drawing our attention to these papers. We now integrate their contributions into our paper and now cite them accordingly.

6- 100,000 schools sounds a big number, but how many schools are there is US? Is there any non-random selection of these schools?

Response: We now provide more detail on the schools in the sample on page 11. Our SafeGraph sample features data on 109,905 public and private schools in December 2020. Given that we have harmonized and comprehensive demographic data for all public schools, we exclude private schools and limit our analysis to the 80,785 public schools in the database. These schools span 12,727 school districts. The total number of public schools in the U.S., according to the National Center for Education Statistics, is 98,469, and the total number of districts is around 13,584. That places our coverage rate of public schools at 82 percent, and coverage rate for districts at 94 percent. Compared to the schools in our SafeGraph data, the 18 percent of schools missing from our dataset have similar average enrollment sizes, shares of homeless students, and free/reduced-price lunch eligibilities, but have students that are slightly more likely to be Black (average of 2.4 percentage points higher), Latino (0.4 percentage points), and Asian (0.7 percentage points).

[Reviewer #3:]

Thanks for giving me the chance to comment on the manuscript “National Database Reveals Large Socio-economic, Geographic, and Demographic Disparities in Exposure to School Closures and Distance Learning in the US” The authors of this study build a school closure and distance learning database that tracks in-person attendance across more than 100,000 schools throughout 2020. This database allows the authors to estimate whether a school is engaged in distance learning based on year-over-year change in in-person attendance for each school. The authors take advantage of this database to describe the socio-economic, geographic, and demographic characteristics of schools and their students that are engaged in distance learning. They find that school closures in October 2020 (the start of the new academic year) are more common in schools with lower pre-COVID education results, measured by math scores in 3rd grade, with higher share of minority’s students, with limited English proficiency, coming from families of lower socioeconomic status and living in single-parent families. In sum, the authors find that, as a result of the pandemic, inequalities in learning outcomes have increased, since studies have shown the negative effect of distance learning on educational performance vis a vis face-to-face learning.

The most important value added of the study is the construction of the database, which gathers very relevant information. The authors are careful to perform three validation checks to ensure that their estimates of schools engaged in distance learning are consistent with alternative data sources.

Also, the descriptive analysis gives relevant information to show how the effects of the COVID-19 pandemic are worsening the pre-COVID inequalities in US education. Indeed, family has always been a mediator between the school and the students, but never as decisive as today. Home is certainly not a school, but in distance learning it has been emulating it. To understand how critical this issue is, it is important to remember that to ensure children's equal rights, formal education has been organized as far as possible from the unequal student's family conditions, offering in exchange, a common experience in time, resources, curriculum, and space for sociability and study. Confining the school at home significantly jeopardizes this elementary principle of equity. Therefore, in the case of the US, it is not only that children from low socioeconomic status families do not attend school, compared to children with more resources, but precisely those children for whom school is more relevant are not having that experience.

Besides putting together the database and provide a descriptive analysis I would like to see a greater use of the data to go beyond descriptive analysis and better understand the consequences of distance learning. It is necessary to provide empirical robust evidence to think about education policies aiming at closing these gaps.

Response: We appreciate the reviewer's comment and suggestion here; however, in line with the judgment from the editors, we have decided not to add new analyses to this study. Our objective instead is to introduce the data, provide the validation tests, and provide high-quality evidence on who is exposed to school closures.

I think it is better to organize the paper explaining first data sources and methods and then the results and their discussion

Response: Per the editorial policy of Nature Human Behavior and recommendations of the editors, we have kept the structure as is but we now include an extra paragraph leading the findings section to preview our data sources and methods first.

[Reviewer #4:]

The study "National Database Reveals Large Socio-Economic, Geographic, and Demographic Disparities in Exposure to School Closures and Distance Learning in the U.S." examines the association between school demographic and socioeconomic characteristics and distance

learning based on school closure proxied by cell phone pings at school locations in over 100,000 schools in the United States. The authors found that school closures are associated with higher shares of racial/ethnic minority students, lower pre-COVID 19 test scores, and other measures of student socioeconomic and academic disadvantage. The study's use of cell phone data to answer an important question is quite novel. I offer several comments below.

Can the authors clarify whether they include private schools in their analysis. If they do include private schools, I suggest the authors testing the sensitivity of their results by focusing only on public schools. Some of the racial/ethnic and socioeconomic disparities may be driven by differences between the enrollment characteristics of public and private schools.

Response: We only include public schools in the analysis. Comprehensive school-level covariate data for private schools is not provided in the Urban Institute Education Data Portal and its underlying datasets. The SafeGraph data do feature information for many private schools, but we do not use the data for these schools given the concerns noted above. We now clarify this in the manuscript in pg. 11-12.

The authors state in a footnote on page 3 that their coverage rate is ``around 77 percent of all schools.' Can the authors describe or characterize the 23 percent of schools not included in the analysis?

Response: We have now added a more detailed breakdown of the schools present within our dataset on pg. 11-12. As noted there, our SafeGraph sample features data on 109,905 public and private schools in December 2020. Given that we have harmonized and comprehensive demographic data for all public schools, we exclude private schools and limit our analysis to the 80,785 public schools in the database. These schools span 12,727 school districts. The total number of public schools in the U.S., according to the National Center for Education Statistics, is 98,469, and the total number of districts is around 13,584. That places our coverage rate of public schools at 82 percent, and coverage rate for districts at 94 percent. Compared to the schools in our SafeGraph data, the 18 percent of schools missing from our dataset have similar average enrollment sizes, shares of homeless students, and free/reduced-price lunch eligibilities, but have students that are slightly more likely to be Black (average of 2.4 percentage points higher), Latino (0.4 percentage points), and Asian (0.7 percentage points). In a sensitivity check, we impute the missing in-person attendance data for schools not in our SafeGraph sample with the mean of other schools in the same district (based on the fact that schools within districts have heavily correlated changes in in-person attendance). In doing so, we reach 93,314 schools for a coverage rate of 95 percent. The sensitivity check does not produce closure rates or socio-economic variance in closure rates that vary meaningfully from our primary analysis (in part due

to the mean imputation). We thus only include the 80,785 schools for which we have observed data in our primary analyses.

* The authors tested the sensitivity of their chosen closure cutoff by running the analyses using a 25 percent decline in attendance in addition to the 50 percent decline used in the main analysis. Why did the authors also not examine a higher cutoff, say, 75 percent?

Response: Our new Figure 6, which compares our SafeGraph data to EdWeek classifications of school closures from September, reveals that a 75 percent cutoff captures only 10 percent of the "fully remote" schools and none of the hybrid/partial or in-person schools. As such, we do not find it a useful cutoff point. We now clarify this in the manuscript on pg. 15-16. Researchers who want to test alternative cutoff points can do so using the data from our public dataset, as we now also include the mean year-over-year changes in in-person attendance for each district (and for each census tract, county, and state).

* Figure 3 examines student enrollment in closed schools as opposed to closed schools. I suggest also including enrollment in Table 2.

Response: Given that the former Figure 3 (now Figure 2) and Table 2 show the same information, we have deleted Table 2 in the revised version of the manuscript. This also allows us to meet the request of other reviewers to condense the findings and avoid repetition.

* Figure 4 - top right graph - should be white students rather than white residents.

Response: We have now fixed this label.

* Many public school distance learning mandates or decisions are made at the school district level. Can the authors either include in their regression or justify why variation between district is not driving results (in a similar way that states may be driving results in the example of Asian students on page 10).

Response: Yes, we believe the reviewer's interpretation is correct: district-level variation is an important driver in geographic dispersion of school closures, particularly in states and counties without jurisdiction-wide mandates. Our exclusion of it in the regression analysis was not meant to signal that districts are unimportant here. But this may point to a larger problem with that analysis: readers may think we are trying to "explain" the disparities we see in exposure to distance learning, when in reality, we are simply assessing whether disparities exist within states and counties, in addition to across states and counties. An added challenge is that these results vary by month (ex: September 2020 estimates look different than December 2020 estimates,

which may look different than estimates in 2021 given ongoing changes in rates of school closures). Taking all of this into account, we decided to delete the figure and analysis and instead focus simply on providing the rich set of descriptives that are present in our other figures. To properly account for within- versus between-jurisdiction variation in closures requires a more detailed analysis and space to assess changes in these patterns by month. The decision to remove this analysis also meets the suggestion of other reviewers to condense our Findings section. Should the editors or reviewers disagree with this decision, we can certainly add the analysis back in, but in our judgment, the study is more focused and concise without it.

References

Yang, T. C., Emily Choi, S. W., & Sun, F. (2020). COVID-19 cases in US counties: roles of racial/ethnic density and residential segregation. Ethnicity & health, 1-11.

[Reviewer 5:]

Thank you for the opportunity to review this novel and important paper. The database provided by the authors is valuable, as are the analyses. However, there are several concerns that need to be addressed before the paper is ready for publication.

1A) The 50% threshold of year-over-year decline in in-person attendance to assign a school to distance learning seems arbitrary and potentially problematic. First, many schools and districts used a hybrid model with half of students attending on any given day to reduce density in schools. This scenario would reduce in-person attendance as measured by phone data by nearly half, but would not accurately indicate distance learning. Attending school in-person half-time allows teachers to come closer to the learning opportunities provided by full in-person attendance.

Response: The reviewer is right that the 50% cutoff used in this study's primary analysis is somewhat arbitrary (why not 25%, 75%, or somewhere in between?), so we have taken two steps to address this concern. First, we have added Figure 6, which uses manually-collected EdWeek school closure classifications (in-person, hybrid/partial, remote) from September to show how our school closure estimates align with their estimates for 900 districts. The results show that 80

percent of school districts with at least a 50 percent decline in in-person attendance (according to our SafeGraph data) are labeled by EW as “fully remote”. The other 20 percent of districts meeting this benchmark are primarily the “hybrid/partial” group. Additionally, 73 percent of all districts that EW classifies as “fully remote” meet the 50 percent cutoff according to our SafeGraph data. The results thus suggest that the 50 percent in-person attendance cutoff effectively captures districts that have explicitly stated that they were fully remote; however, it does not capture all such districts (a minority of remote schools fall below the cutoff), nor does it exclusively capture such schools (some districts meeting this benchmark are classified as “hybrid/partial”). The evidence also suggests that a 25 percent year-over-year change benchmark captures the majority of districts in each of the three categories, whereas a 75 percent year-over-year change benchmark captures very few schools in general. Second, in our public database, we also present estimates based on alternative cutoffs (25%, 50%, and 75%), as well as the mean of the continuous measure of the decline in in-person attendance to nonetheless provide researchers the full set of options in using our database. This increases transparency in the results and allows researchers who use our data to test their findings using multiple cutoff points, or simply the mean year-over-over change.

1B) Second, error in assignment based on the 50% threshold is likely correlated with the percent of students eligible for free lunch. Schools generally serve as the free lunch pick-up location for students attending school remotely. Schools with high poverty rates would be under-represented in those assigned to distance learning.

Response: The reviewer is right that if families still visit schools to grab their lunches (and then return home), this may register in our data as a visit to the school and lead us to undercount the extent of school closures among such schools. We acknowledge this potential limitation on pg. 16-17 in our *Limitations* section. We also search for evidence of the pattern that the reviewer suggests. In the figure below, we document the association of the share of students who are FRPL-eligible versus year-over-year change in the average “dwell time”, or the average duration of visits of mobile devices to a school in a month, among schools that do not meet our benchmark of closed or mostly closed. We emphasize in advance that we have not been able to validate the “dwell time” indicator and we do not use it for the analyses in our study. We use it here simply as one potential mechanism to test the relationship that the reviewer suggests.

Figure: Binned scatterplot of FRPL eligibility (Y-axis) by dwell time decile (X-axis) among schools not identified as closed or mostly closed

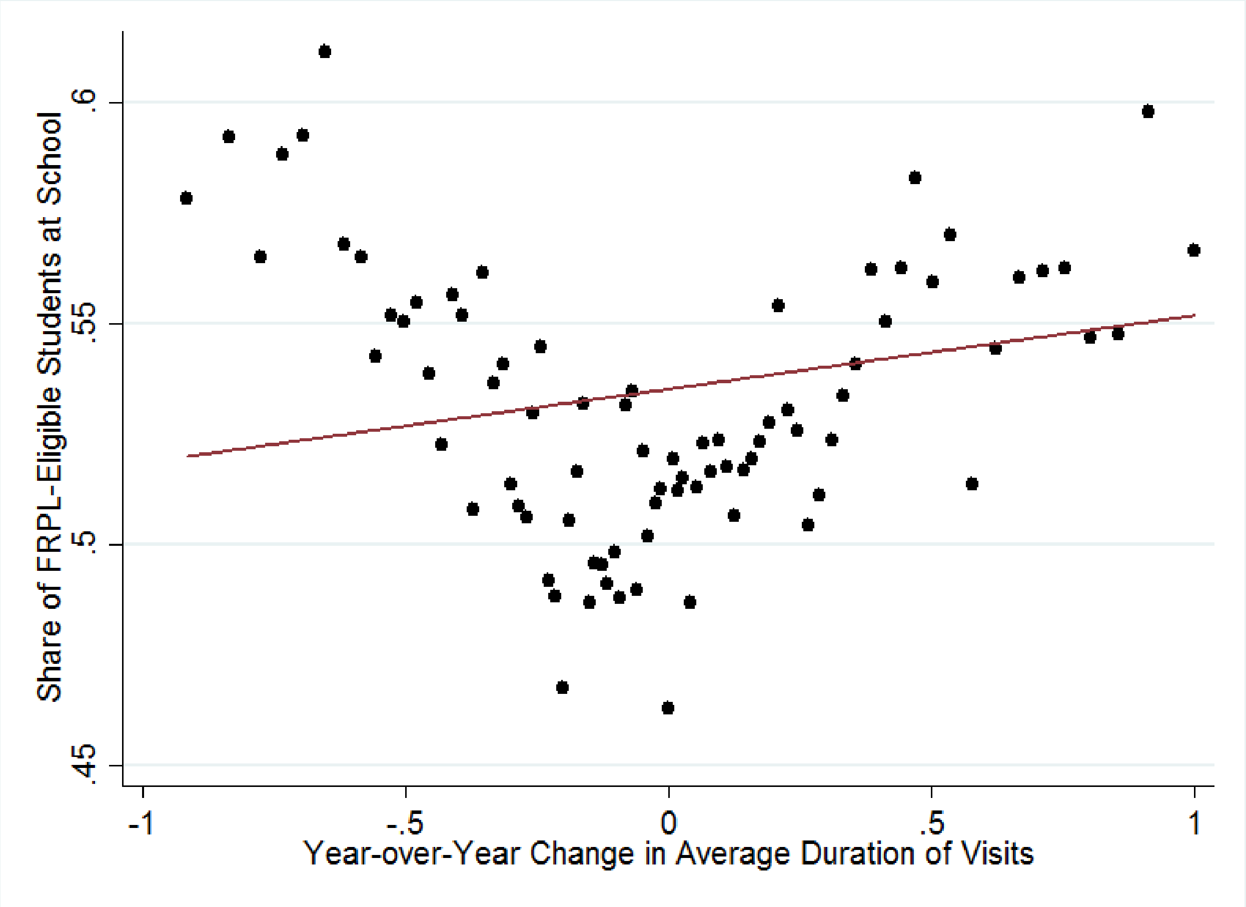

The figure suggests a positive correlation between change in dwell time and FRPL eligibility, suggesting that it is not the case, on average, that high-FPRL schools are experiencing a greater increase in quick pickups of food before returning home. However, the figure clearly shows a V-shape, in which many points on the far left of the figure suggest that schools seeing the largest decline in dwell time do have FRPL-eligibility rates that are higher than average. As a sensitivity check, we re-examine our results while classifying schools that see a 50% or greater decline in average dwell time (the left side of the figure above) as “closed” even if they do not register a 50% decline in in-person attendance. Such schools are the likeliest potential candidates to fall into the measurement error concern that the reviewer describes. When making this change, the total share of students in “closed” schools in December increases from 62.3% to 65.9%. However, the *inequalities* in exposure to school closures do not meaningfully change. Consider the new figure below, which presents a revised version of Figure 3 within our paper, but now with the new measure of “closed” schools that includes those with 50% declines in average dwell

time. This figure is nearly identical to the original version, except the levels of school closures (Y-axis) tend to be slightly higher.

Figure: Revised version of Figure 3 but with inclusion of schools experiencing a 50%+ decline in average dwell time as closed, regardless of percent change in attendance, December 2020

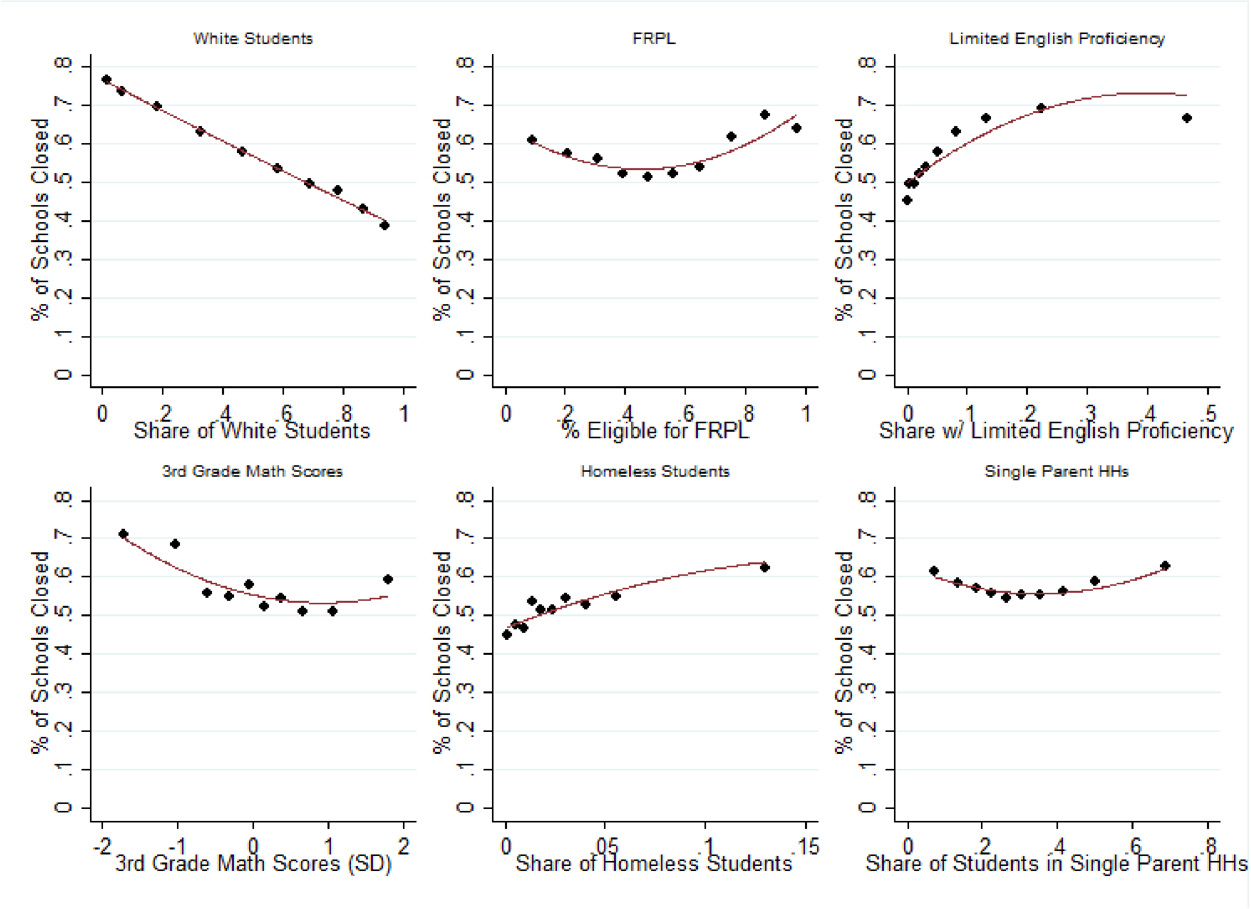

Put simply, while we acknowledge the reviewer's concern and mention it within our study, we do not find sufficient evidence within our data that potential measurement error related to school closures and FRPL-eligibility meaningfully affects our findings. We do not currently include the sensitivity check in our revised manuscript, as doing so requires two extra figures and a discussion of the "dwell time" metric, which we are unable to validate. However, we are happy to include the analysis above in the final version at the request of the editors or reviewers.

1C) The authors should take more advantage of the information provided in the continuous measure of percent decline in attendance compared to the previous year. For example, the authors could identify a representative group of schools that used a hybrid attendance model, learn what their year-over-year decline in attendance was, and create a range to indicate likely hybrid attendance. The authors could use a similar procedure to create a range for likely full distance learning schools. Alternatively, the authors could use the continuous measure of decline in attendance to quantify the relationship between attendance and their axes of inequality (e.g., race, ethnicity, free/reduced price lunch, ESL students).

Response: We agree with the reviewer and have taken several steps to address these concerns. First, we now include the continuous measure of the mean change in in-person attendance into our public database. This allows researchers who prefer that measure over the binary estimates to use it accordingly. Second, our new Figure 6 gets at the reviewer's suggestion to compare percent decline in in-person visits (according to SafeGraph) to classifications of in-person, hybrid/partial, or remote learning (according to EdWeek classifications of 900 districts in September). Figure 6 makes clear that the group we can identify with the most precision is the fully-remote classification. Though it would be ideal to find a precise range of values that matches each classification (hybrid/partial vs. open, for example), the data simply do not support such an approach. Instead, the best we can do is measure the "fully remote" group with a high degree of certainty. Third, with respect to comparing the continuous measure of change to our socio-economic indicators: the results just are not much different from what we already show in the binned scatterplot of Figure 3 (our mean of school closures across the distribution of our socio-economic indicators). See the figure below, for example:

Figure: Revised version of Figure 3 with percent decline in in-person attendance as Y-axis rather than share of schools closed (binned scatterplot)

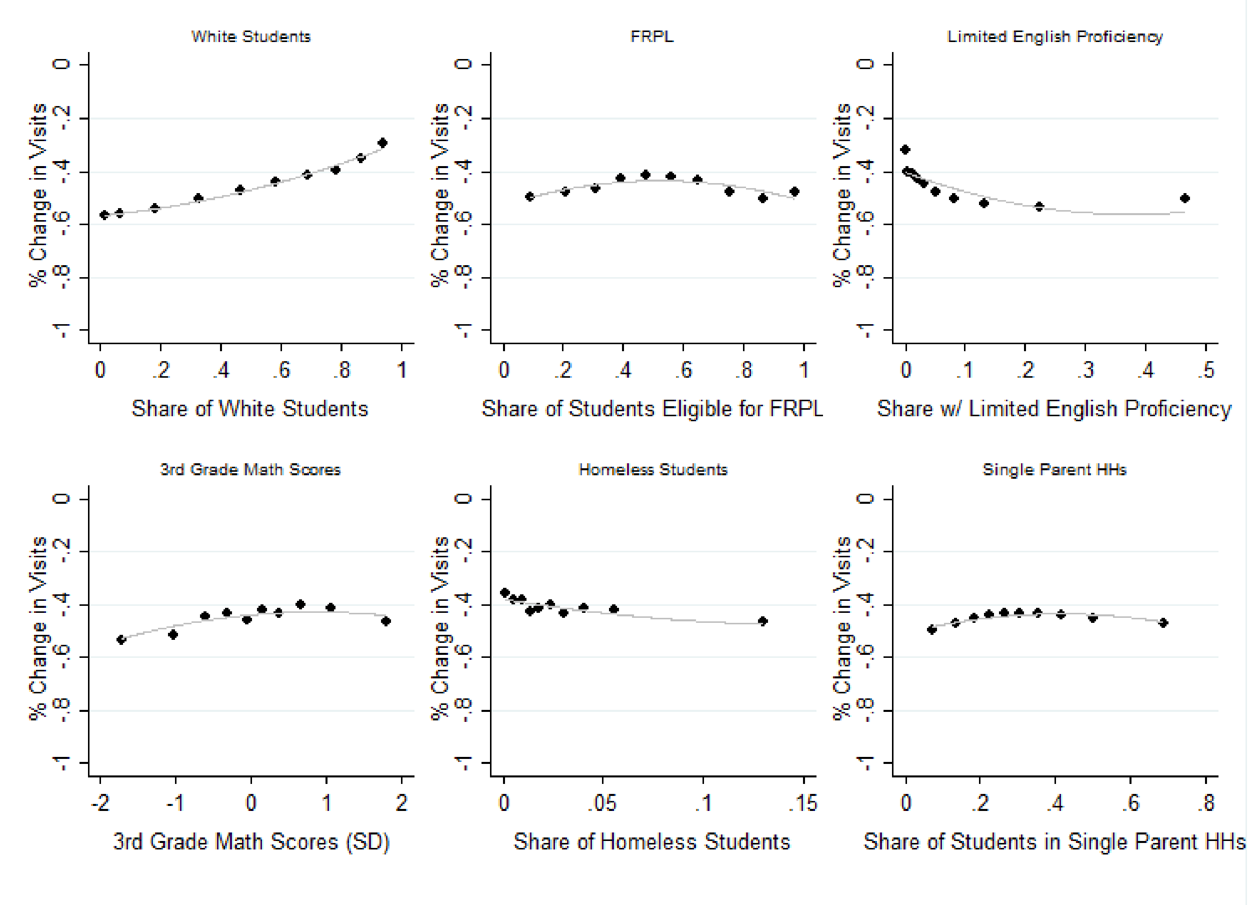

This figure is simply the inverse of the current Figure 3, which plots the share of schools closed on the Y-axis instead. Thus, shifting the Y-axis from the share of schools closed (with the 50% decline cutoff) to the continuous measure of year-over-year decline does not add new information in this particular case. We thus do not include the extra figure above into our manuscript.

2) The authors should address potential correlation between error in assignment to distance learning and the percent of students eligible for free lunch. It is difficult or misleading to compare their measures of inequality without addressing that bias.

Response: We now acknowledge this limitation on pg. 16-17. But, as we note in our response above, we do not find strong evidence of the reviewer's suggested pattern with our available data, and we lack additional tools to investigate the potential error that the reviewer is suggesting.

3) Internet access is another important axis of inequality that would be valuable to examine in this paper.

Response: We agree that this is an important aspect to study, but we do not have school- or district-specific data on internet access. We assessed the possibility of using the American Community Survey to gain internet access data at a local level, but the ACS still features a lot of missing data for very small geographic units (we have data coverage from the ACS for around 60 percent of the schools in our study, unlike our other indicators, for which we have full coverage). Though we cannot address internet access in the present study, we now note it as an important aspect to investigate further should higher-quality data at the local level become available.

4) The authors frame the study as measuring inequality in learning opportunities alone. However, school closure may be an important response to higher mortality in those areas. The authors could improve the paper by noting that the inequality in learning opportunities may represent a tradeoff with inequality in health/mortality risk.

Response: We agree and acknowledge this on pg. 9. There, we write: "One should *not* infer from our findings that school closures, despite their estimated effects on educational performance, are unwarranted or that they necessarily do more harm than good. To the extent that schools are fertile grounds for the transmission of COVID-19, school closures may save lives, particularly in communities with more racial/ethnic minorities and/or lower incomes (the groups that this study finds are more likely to be exposed to school closures). The decision to turn to distance learning is undoubtedly difficult and is fraught with trade-offs; this study is not designed to address, and takes no position on, whether the costs of distance learning outweigh the benefits."

Decision Letter, first revision:

15th February 2021

Dear Dr. Parolin,

Thank you for your patience as we've prepared the guidelines for final submission of your Nature Human Behaviour manuscript, "Large Socio-Economic, Geographic, and Demographic Disparities Exist in Exposure to School Closures" (NATHUMBEHAV-201213551A). Please carefully follow the step-by-

step instructions provided in the personalised checklist attached, to ensure that your revised manuscript can be swiftly handed over to our production team.

We hope to receive your revised paper, with all of the requested files and forms, within 10 days. If you anticipate delays, we would be grateful if you could contact us to provide us with an estimate regarding when you will submit these files.

When you upload your final materials, please include a point-by-point response to any remaining reviewer comments.

If you have not done so already, please alert us to any related manuscripts from your group that are under consideration or in press at other journals, or are being written up for submission to other journals (see: https://www.nature.com/nature-research/editorial-policies/plagiarism#policy-on-duplicate-publication for details).

Nature Human Behaviour offers a Transparent Peer Review option for new original research manuscripts submitted after December 1st, 2019. As part of this initiative, we encourage our authors to support increased transparency into the peer review process by agreeing to have the reviewer comments, author rebuttal letters, and editorial decision letters published as a Supplementary item. When you submit your final files please clearly state in your cover letter whether or not you would like to participate in this initiative. Please note that failure to state your preference will result in delays in accepting your manuscript for publication.

In recognition of the time and expertise our reviewers provide to Nature Human Behaviour's editorial process, we would like to formally acknowledge their contribution to the external peer review of your manuscript entitled "Large Socio-Economic, Geographic, and Demographic Disparities Exist in Exposure to School Closures". For those reviewers who give their assent, we will be publishing their names alongside the published article.

Cover suggestions

As you prepare your final files we encourage you to consider whether you have any images or illustrations that may be appropriate for use on the cover of Nature Human Behaviour.

Covers should be both aesthetically appealing and scientifically relevant, and should be supplied at the best quality available. Due to the prominence of these images, we do not generally select images featuring faces, children, text, graphs, schematic drawings, or collages on our covers.

We accept TIFF, JPEG, PNG or PSD file formats (a layered PSD file would be ideal), and the image should be at least 300ppi resolution (preferably 600-1200 ppi), in CMYK colour mode.

If your image is selected, we may also use it on the journal website as a banner image, and may need to make artistic alterations to fit our journal style.

Please submit your suggestions, clearly labeled, along with your final files. We'll be in touch if more information is needed.

Nature Human Behaviour has now transitioned to a unified Rights Collection system which will allow our Author Services team to quickly and easily collect the rights and permissions required to publish your work. Approximately 10 days after your paper is formally accepted, you will receive an email in providing you with a link to complete the grant of rights. If your paper is eligible for Open Access, our Author Services team will also be in touch regarding any additional information that may be required to arrange payment for your article.

Please note that you will not receive your proofs until the publishing agreement has been received through our system.

For information regarding our different publishing models please see our Transformative Journals page. If you have any questions about costs, Open Access requirements, or our legal forms, please contact ASJournals@springernature.com.

Please use the following link for uploading these materials:
[REDACTED]

If you have any further questions, please feel free to contact me.

Best regards,

Charlotte Payne
Editor
Nature Human Behaviour

Reviewer #1:
Remarks to the Author:
The authors have done an excellent job responding to the reviews. The manuscript and the database are valuable resources.

2 minor quibbles:
1. I'm confused by the response to the suggestion to use SEDA rather than opportunity atlas data. OA has data from 2013 at the tract level; SEDA has more recent data at the district and county level -- which seems more appropriate for the database here (tracts don't map onto districts easily). the concerns about missing data in the text refer to EDFacts data -- which does have a lot of missing/ranged data; but SEDA does not.

2. I think ROC plots would be more informative than the CDF plots in figure 6. ROC plots are better for determining the best location for a binary classification rule than the CDF plots, but that's not a major concern.

Thank you for creating this valuable resource.

Reviewer #2:
Remarks to the Author:
I read the new version and the responses to my concerns and questions, and I appreciate the way they were addressed. Thus, I am ok with the new version. In my opinion, it is ready to be published.

Reviewer #3:
Remarks to the Author:
The authors responded adequately to all the referees' comments and the new version of the article is ready for acceptance.

Reviewer #4:
Remarks to the Author:
The authors have carefully addressed all of my comments and concerns. I believe this paper will provide a novel contribution to the growing literature examining the stratified impacts of COVID-19.

Reviewer #5:
Remarks to the Author:
The authors have done an excellent job of addressing all of the points raised. I applaud the authors for their careful revision and their valuable contribution to knowledge.

Author Rebuttal, first revision:

Response to Reviewers: We again appreciate the responses from the five reviewers. Their suggestions in the first round of revision contributed to a much-improved paper. Below, the five reviewers express that the paper is ready for publication. Per suggestions from the Nature Human Behavior editors in the prior round of reviews, we have maintained the current OA education data in the manuscript, but we will explore updates from SEDA in future work in light of Reviewer's 1 comment below. Many thanks again to the reviewers and editors for a useful and timely revision process.

Reviewer #1: The authors have done an excellent job responding to the reviews. The manuscript and the database are valuable resources.

2 minor quibbles:

1. I'm confused by the response to the suggestion to use SEDA rather than opportunity atlas data. OA has data from 2013 at the tract level; SEDA has more recent data at the district and county level -- which seems more appropriate for the database here (tracts don't map onto districts easily). the concerns about missing data in the text refer to EDFacts data -- which does have a lot of missing/ranged data; but SEDA does not.

2. I think ROC plots would be more informative than the CDF plots in figure 6. ROC plots are better for determining the best location for a binary classification rule than the CDF plots, but that's not a major concern.

Thank you for creating this valuable resource.

Reviewer #2: I read the new version and the responses to my concerns and questions, and I appreciate the way they were addressed. Thus, I am ok with the new version. In my opinion, it is ready to be published.

Reviewer #3: The authors responded adequately to all the referees' comments and the new version of the article is ready for acceptance.

Reviewer #4: The authors have carefully addressed all of my comments and concerns. I believe this paper will provide a novel contribution to the growing literature examining the stratified impacts of COVID-19.

Reviewer #5: The authors have done an excellent job of addressing all of the points raised. I applaud the authors for their careful revision and their valuable contribution to knowledge.

Final Decision Letter:

Dear Dr Parolin,

We are pleased to inform you that your Resource "Large Socio-Economic, Geographic, and Demographic Disparities Exist in Exposure to School Closures", has now been accepted for publication in Nature Human Behaviour.

Before your manuscript is typeset, we will edit the text to ensure it is intelligible to our wide readership and conforms to house style. We look particularly carefully at the titles of all papers to ensure that they are relatively brief and understandable.

Once your manuscript is typeset and you have completed the appropriate grant of rights, you will

receive a link to your electronic proof via email with a request to make any corrections within 48 hours. If, when you receive your proof, you cannot meet this deadline, please inform us at rjsproduction@springernature.com immediately. Once your paper has been scheduled for online publication, the Nature press office will be in touch to confirm the details.

Acceptance of your manuscript is conditional on all authors' agreement with our publication policies (see http://www.nature.com/nathumbehav/info/gta). In particular your manuscript must not be published elsewhere and there must be no announcement of the work to any media outlet until the publication date (the day on which it is uploaded onto our web site).

Nature Human Behaviour is a Transformative journal and offers an immediate open access option through payment of an article-processing charge (APC) for papers submitted after 1 January, 2021. In the event that authors choose to publish under the subscription model, Nature Research allows authors to self-archive the accepted manuscript (the version post-peer review, but prior to copy-editing and typesetting) on their own personal website and/or in an institutional or funder repository where it can be made publicly accessible 6 months after first publication, in accordance with our self-archiving policy. Please review our self-archviing policy for more information.

Several funders require deposition the accepted manuscript (AM) to PubMed Central or Europe PubMed Central. To enable compliance with these requirements, Nature Research therefore offers a free manuscript deposition service for original research papers supported by a number of PMC/EPMC participating funders. If you do not choose to publish immediate open access, we can deposit the accepted manuscript in PMC/Europe PMC on your behalf, if you authorise us to do so.

If you have posted a preprint on any preprint server, please ensure that the preprint details are updated with a publication reference, including the DOI and a URL to the published version of the article on the journal website.

An online order form for reprints of your paper is available at https://www.nature.com/reprints/author-reprints.html. All co-authors, authors' institutions and authors' funding agencies can order reprints using the form appropriate to their geographical region.

We welcome the submission of potential cover material (including a short caption of around 40 words) related to your manuscript; suggestions should be sent to Nature Human Behaviour as electronic files (the image should be 300 dpi at 210 x 297 mm in either TIFF or JPEG format). Please note that such pictures should be selected more for their aesthetic appeal than for their scientific content, and that colour images work better than black and white or grayscale images. Please do not try to design a cover with the Nature Human Behaviour logo etc., and please do not submit composites of images related to your work. I am sure you will understand that we cannot make any promise as to whether any of your suggestions might be selected for the cover of the journal.

You can now use a single sign-on for all your accounts, view the status of all your manuscript submissions and reviews, access usage statistics for your published articles and download a record of

your refereeing activity for the Nature journals.

To assist our authors in disseminating their research to the broader community, our SharedIt initiative provides you with a unique shareable link that will allow anyone (with or without a subscription) to read the published article. Recipients of the link with a subscription will also be able to download and print the PDF.

As soon as your article is published, you will receive an automated email with your shareable link.

In approximately 10 business days you will receive an email with a link to choose the appropriate publishing options for your paper and our Author Services team will be in touch regarding any additional information that may be required.

You will not receive your proofs until the publishing agreement has been received through our system.

If you have any questions about our publishing options, costs, Open Access requirements, or our legal forms, please contact ASJournals@springernature.com

We look forward to publishing your paper.

With best regards,

Charlotte Payne
Editor
Nature Human Behaviour

P.S. Click on the following link if you would like to recommend Nature Human Behaviour to your librarian http://www.nature.com/subscriptions/recommend.html#forms

** Visit the Springer Nature Editorial and Publishing website at www.springernature.com/editorial-and-publishing-jobs for more information about our career opportunities. If you have any questions please click here.**
